# Photoluminescent organisms: how to make fungi glow through biointegration with lanthanide metal-organic frameworks

**DOI:** 10.1038/s41598-019-43835-x

**Published:** 2019-05-13

**Authors:** Jeferson Rosário, Leonis L. da Luz, Regina Geris, Jéssica G. S. Ramalho, Antônio F. da Silva, Severino Alves Júnior, Marcos Malta

**Affiliations:** 1 Institute of Chemistry, Federal University of Bahia, Campus Ondina, Salvador, BA Brazil; 2 Department of Fundamental Chemistry, Federal University of Pernambuco, Cidade Universitária, Recife, PE Brazil; 3 Institute of Physics, Federal University of Bahia, Campus Ondina, Salvador, BA Brazil

**Keywords:** Metal-organic frameworks, Biomaterials - cells

## Abstract

We show that filamentous fungi can emit green or red light after the accumulation of particulate lanthanide metal-organic frameworks over the cell wall. These new biohybrids present photoluminescence properties that are unaffected by the components of the cell wall. In addition, the fungal cells internalise lanthanide metal-organic framework particles, storing them into organelles, thereby making these materials promising for applications in living imaging studies.

## Introduction

Bioluminescence is a fascinating characteristic that some organisms present in emitting visible light through a biochemical reaction^1^. Typically, biological systems produce light through the oxidation of light-emitting substrates, known as luciferins, in conjunction with enzymes, known as luciferases^2^. Recently, Purtov and co-workers elucidated the chemical mechanism of bioluminescence in fungal species through the identification of the luciferin, 3-hydroxyhispidin^3^. Furthermore, it has been proposed that the bioluminescence of some fungal species plays an important role in biological functions. For instance, Oliveira and co-workers have found that light emission by *Neonothopanus gardneri* mushrooms obeys a temperature compensated circadian rhythm^4^. Consequently, as the fungus maximises light emission at night, it can attract several types of insects that are responsible for enhancing fungal propagation through spore dispersal. Nevertheless, it is relatively rare to find fungal species with a luminous ability. From Hawksworth’s estimation of 1.5 million fungi species on Earth, there are only 71 species (less than 0.005%) that have been described as luminescent^5,6^.

Converting non-luminescent microorganisms into glowing ones could provide them with additional functions beyond their original purposes, therefore acting as a huge biotechnological potential that can be applied in areas, such as photo- and biocatalysis and sensing. In addition, luminescent microorganisms hold great promise for applications in *in vivo* imaging that improve the observation of fungal morphology and structure, assisting in the elucidation of hyphae physiology. A promising strategy to produce luminous microorganisms lies in the integration of fungal cells with photoluminescent materials, such as fluorescent conjugated polymers or semiconducting quantum dots. For instance, Wang’s group developed luminescent conducting polymers that are self-assembled on filamentous fungi through electrostatic/hydrophobic interactions^7^. The authors found that water-soluble polythiophene backbones can only be accumulated on the fungal surface, yielding luminescent cells. Alternatively, Rispail and co-workers observed that hydrophilic CdSe/ZnS quantum dots functionalised with 3-mercaptopropanoic acid are internalised by *Fusarium oxysporium* fungus, which can be used for the rapid and sensitive detection of this phytopathogen^8^.

At present, there is considerable interest in the artificial introduction of abiotic materials in the structure of living organisms to form biohybrid entities^9–15^. The expectation is that these biohybrids can enable new or have improved properties when compared to the native counterparts. For instance, Giraldo and co-workers reported that the integration of single-walled carbon nanotubes (SWCNTs) within plant chloroplasts led to the rise of the solar energy conversion for both *in vivo* (leaves) and *ex vivo* ones (extracted plant chloroplasts)^16^. The authors suggested that an enhancement in the photosynthetic activity of chloroplasts may occur due to the change in absorption profile by SWCNTs, resulting in light capture in the range of ultraviolet, green and near-infrared spectra. In another example, the integration of the electroactive conducting polymer poly(3,4-ethylenedioxythiophene) by the vascular network of *Rosa floribunda* (garden rose), reported by Stavrinidou and co-workers, provided a new strategy for developing plants with electronic functionalities, such as transistor modulation, digital logic function and electronic conductivity^12^.

Among a myriad of functional materials suitable for biohybrid production, metal-organic frameworks (MOFs) are rapidly emerging as an exceptional class of porous materials. MOFs can be defined as a coordination network built by a large number of metal ions, metal-containing clusters and organic ligands^17–19^. Compared to other porous materials, such as zeolites, active carbon or mesoporous silica, MOFs exhibit better chemical flexibility due to the diverse functional groups of their frameworks^20–23^. These characteristics make MOFs one of the most promising materials for potential applications beyond the traditional areas of porous materials, such as molecular storage, separation and catalysis^19,20,24–27^. Luminescent lanthanide metal-organic frameworks (Ln-MOFs, where Ln = Eu^3+^, Tb^3+^, Gd^3+^ or Nd^3+^) were successfully used as a platform for the production of white-light emitting materials^28^. In addition, Ln-MOFs have been printed as photoluminescent inks onto plastic and paper foils to produce invisible security labelling/encoding^29^. Of particular interest, MOF-eukaryotic cell (yeasts) biohybrids have been described as promising biosystems because they can survive in harsh conditions when compared to native cells^30,31^. Initially, the production of a MOF exoskeleton over single cells occurs through the concentration of MOF precursors at the cell wall. Subsequently, crystallisation takes place, recovering the whole cell with an artificial carapace. For instance, Liang *et al*. reported the preparation of a crystalline MOF protective coating for living cells^30^. The main characteristic of this biosystem is that the MOF exoskeleton controls the molecular trafficking to the cell cytoplasm and prevents cell multiplication by inducing an artificial hibernation state. Then, after removal of the MOF shell, the cells can recover full functionality.

Here, we report our findings of the ability of common filamentous fungi to accumulate Ln-MOFs over their cell walls, thereby forming new classes of luminescent biohybrid cells. When microorganisms are cultivated in solutions containing a carbon source and discrete particulate Ln-MOFs, they deposit these non-biological objects over mycelia during the physiological process, forming a robust layer on the cellular wall of the fungi. Moreover, we have also observed the preferential accumulation of Ln-MOFs on fungal organelles, demonstrating their ability for the internalisation of luminescent particles. The implications of these observations could be very relevant to various areas of chemistry, physics and biology. First, we demonstrate that the integration of Ln-MOFs onto fungal mycelia is a quite general process, producing microorganisms that emit visible light when excited with ultraviolet radiation (i.e. photoluminescence). Second, the utilisation of fungi as scaffolds to reach hybrid structures with complex morphologies presents clear advantages in the development of new materials, such as size uniformity, broad bioavailability, renewability, low costs and so on. Finally, the modification of the cell surface with Ln-MOFs also led to an uptake of luminescent particles to the cytoplasmic region, making this strategy potentially useful for living imaging applications in filamentous fungi.

## Results and Discussion

Filamentous fungi are morphologically complex microorganisms, in which the primary structure of growth consists of a tubular filament known as a hypha. Different from single-cell organisms, such as bacteria and yeasts, filamentous fungi develop through the elongation of the hypha at the tip, to explore different regions in the search for nutrients. Currently, the only method for the deposition of artificial materials on filamentous fungi consists of the cultivation of fragments of mycelia or fungal spores in the presence of particulate nanomaterials. That is, the presence of pre-formed nanomaterials dispersed in a solution is a *sine qua non* condition to form an exoskeleton over the tubular cells. In a typical experiment, fungal spores of *Phialomyces macrosporus*, *Trichoderma* sp. or *Aspergillus niger* were inoculated in a sterile solution containing glucose and a previously sonicated Ln-MOF dispersion. Tb- and Eu-MOFs were prepared using mellitic acid as the organic linker and their corresponding metal salt, Ln(NO_3_)_3_, as discussed in the ESI. Mellitic acid (1,2,3,4,5,6-benzenehexacarboxilic acid) was chosen in our experiments to prepare the Ln-MOFs, in order to facilitate the interaction with the fungal wall through intermolecular hydrogen bonds. Fig. S1 depicts the coordination environment of mellitate and Ln^3+^ ions in the Ln-MOFs and their structure along the b axis. After sonication, an aqueous dispersion containing particulate Ln-MOFs had a measured size varying from 70 to 900 nm (ESI, Figs S2 and S3) and a negatively charged surface estimated from the zeta potential equal to −30 mV. Fungi were cultured for two weeks in the dark in ambient conditions. After growth of the microorganisms, fungal mycelia decorated with Ln-MOFs were copiously rinsed with deionised water and divided in two sets for analysis. In the first set of samples, fungal mycelia were dehydrated with ethanol and dried using critical point drying for further physicochemical characterisation. In the second set, mycelial tissue was stored in deionised water at 4 °C for fluorescence microscopy analysis.

**Figure 1:**
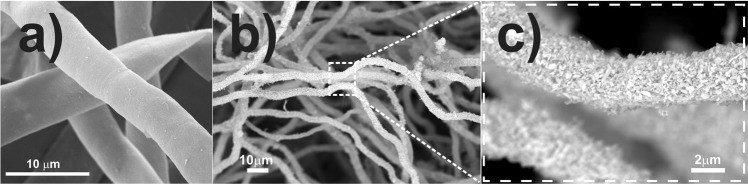
SEM micrographs of native *A*. *niger* (**a**) and the *A*. *niger*/Tb-MOF biohybrid (**b**,**c**).

Figure 1 shows representative scanning electron microscopy (SEM) micrographs highlighting the morphological differences between fungal hyphae cultivated in the absence of and exposed to a nutritive solution containing Tb-MOF particles. Native *A*. *niger* hyphae (Fig. 1(a)) present a tubular morphology with a homogenous smooth surface. Alternatively, the presence of any particulate material in solution leads to drastic changes in the microorganism ultrastructure, with a controlled overall deposition of block-like Ln-MOFs on the hyphae wall (Fig. 1(b,c)). The SEM images reveal that particulate Ln-MOFs are accumulated uniformly along the whole tubular cell, with no favoured sites for deposition of the abiological material. Higher magnification images (ESI, Fig. S4) show that Ln-MOFs are found to be entrapped in a biopolymer matrix, which firmly binds these materials over the fungal surface.

From a chemical perspective, the mechanism of deposition of abiotic particles over filamentous fungi is unknown and is still under debate. In principle, there is a clear link between fungal growth and the deposition of nanoparticles. The best description of the integration of nanomaterial on the cell wall structure is the nutrition driven deposition presented by Sugunan and co-workers^32^. In this article, the authors cultured fungi species in colloidal solutions containing glutamate-stabilised gold nanoparticles. According to the experiments, the hyphal growth coincides with the deposition of particles around the fungal cell. It was suggested that the fungal growth consumes glutamate ions (which serve as capping agents for nanoparticles and a carbon source for the microorganisms), following the depletion of these ions in the vicinity of fungal hyphae. Thus, the decline of glutamate ions due to the fungal metabolism could cause destabilisation of the charge arrangement on the nanoparticle surface, inducing the agglomeration and coating of particles over hypha. Regarding the interaction microorganism/nanomaterial, we later proposed a scenario based on the cultivation of fungi in solutions of citrate-stabilised gold nanoparticles^33^. Transmission electron microscopy studies of *P*. *macrosporus*/Au-NP biohybrids revealed that the metal nanoparticles remain isolated from each other, probably stabilised by steric effects of major components of the fungal cell. Therefore, the deposition of nanomaterials may be attributed to the substitution of the weak interaction of citrate-NPs in solution with a stronger bind between functional groups of the cell wall and the nanoparticles. In addition, other aspects should be considered to explain the deposition of nanomaterials onto fungal hyphae. For instance, Zhu *et al*. emphasised the importance of extracellular polymeric substances (EPS) as being beneficial to the process of deposition of nanoparticles at the fungal cell wall^34^. EPS are biopolymers constituted by proteins and carbohydrates secreted by the microorganisms, which perform particularly important biological processes as assistance to the colonisation of new niches, cellular adhesion in living and inanimate substrates, as well as function as a protective layer around the cell^35,36^. Thus, it is plausible to suppose that EPS secreted by filamentous fungi could functionalise the surface of the suspended solids in solution, enriching its surface with carbonyl and hydroxyl groups, facilitating the adherence of nanoparticles on the fungal wall.

**Figure 2:**
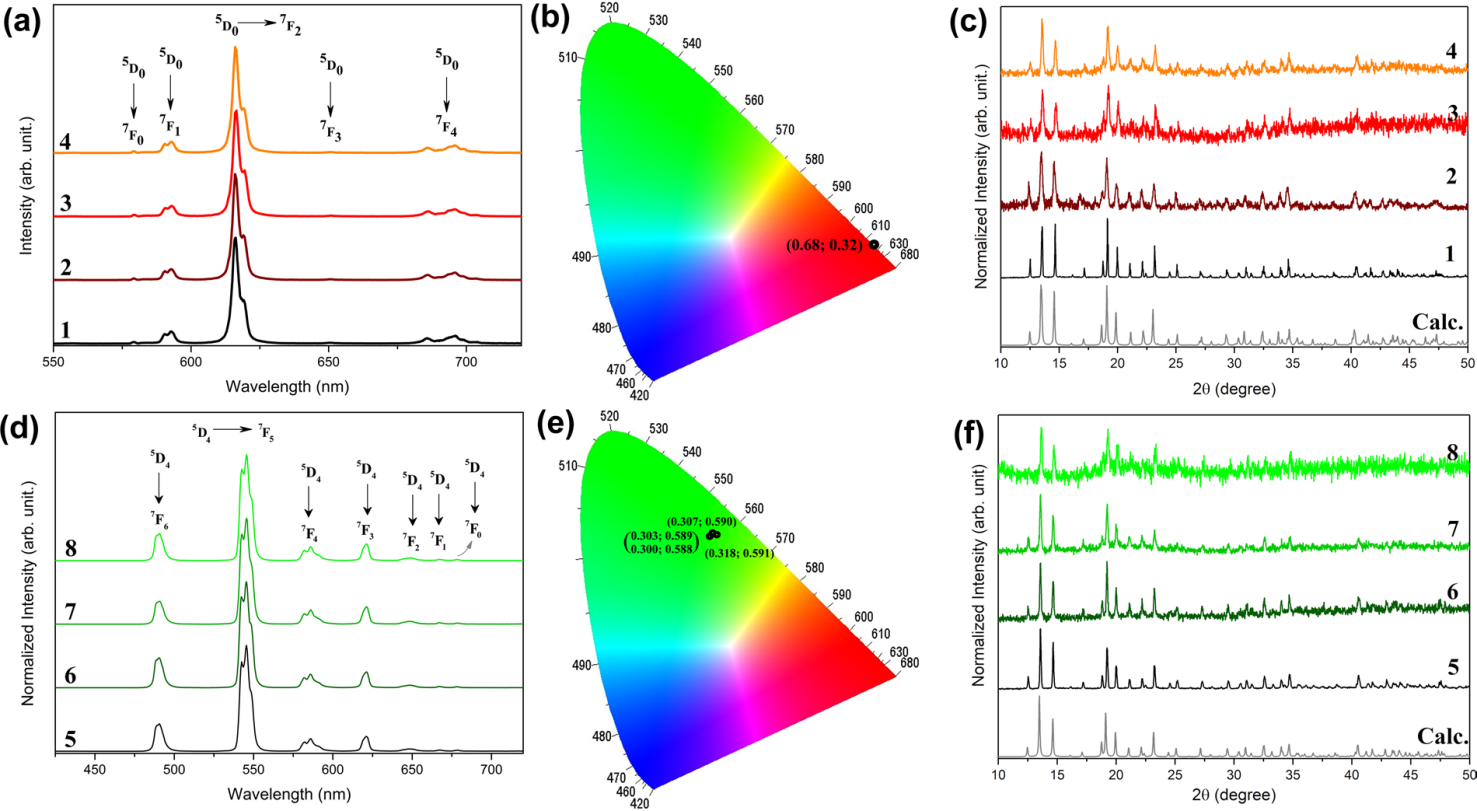
Emission spectra (λEx = 312 nm) and CIE chromaticity diagram containing the photoluminescence colours corresponding to each emission spectra of the Eu-MOF (**a**,**b**) and Tb-MOF (**d**,**e**) based materials, and experimentally calculated PXRD diffraction patterns (**c**,**f**) of Eu-MOF (1), *A*. *niger*/Eu-MOF (2), *Trichoderma sp*./Eu-MOF (3), *P*. *macrosporus*/Eu-MOF (4), Tb-MOF (5), *A*. *niger*/Tb-MOF (6), *Trichoderma sp*./Tb-MOF (7) and *P*. *macrosporus*/Tb-MOF(8).

A complementary physicochemical characterisation was carried out to confirm the integration of Ln-MOFs on microorganisms through energy-dispersive X-ray (EDX) analysis (ESI, Fig. S5) and X-ray diffraction (XRD). Moreover, photoluminescence was used to measure the eventual interaction and/or decomposition of the Ln-MOFs near to the interface between fungal hyphae and Tb- and Eu-MOFs, since the photoluminescence of trivalent lanthanide ions is extremely dependent on the symmetry of the first coordination sphere^37,38^. The XRD patterns of the *A*. *niger*/Ln-MOF, *Trichoderma* sp./Ln-MOF and *P*. *macrosporus*/Ln-MOF biohybrids indicate the presence of more intense diffraction peaks indexed to Ln-MOF structures at 13.4° (hkl; 020), 14.5° (101), 19.1° (12-1), 20.0° (121) and 23.0° (022) (Fig. 2(c–f))^28,29,39^.

Photoluminescence measurements of the critical point dried biohybrids were carried out at room temperature. Figure 2(a,d) show the emission spectra obtained upon excitation at 312 nm $({\pi }^{\ast }\leftarrow \pi )$ of the mellitate ligand for Ln-MOFs and fungi/Ln-MOF biohybrids. These emission spectra display narrow bands characteristic of Eu^3+^ (${}^{5}D_{0}\to {}^{7}F_{J}$; J = 0, 1, 2, 3 and 4) and Tb^3+^ (${}^{5}D_{4}\to {}^{7}F_{J}$; J = 0, 1, 2, 3, 4, 5 and 6) ions for Eu-MOFs and fungi/Eu-MOFs, and Tb-MOFs and fungi/Tb-MOFs, respectively. In both cases, the suppression of ligand emission is suggestive of the effective energy transfer from ligand absorption to f levels of Eu^3+^ and Tb^3+^ ions. This behaviour leads to a photoluminescence colour and respective chromaticity coordinates (x; y) in the International de l’Eclairage (CIE) diagram in the red for Eu-MOF (0.680, 0.319), *A*. *niger*/Eu-MOFs (0.680, 0.319), *Trichoderma* sp./Eu-MOFs (0.681, 0.318) and *P*. *macrosporus*/Eu-MOFs (0.681, 0.318); and green for the Tb-MOFs (0.303, 0.589), *A*. *niger*/Tb-MOFs (0.300, 0.588), *Trichoderma* sp./Tb-MOFs (0.318, 0.591) and *P*. *macrosporus*/Tb-MOFs (0.307, 0.590), as illustrated by the CIE chromaticity diagrams in Fig. 2(b,e)^40^.

The relative intensities and the number of Stark components are dependent upon the extent to which the (2 J + 1) degeneracy is removed by the symmetry of the first coordination sphere. Thus, they can be used as a probe of symmetry sites in europium-based compounds^37,38^. Thereby, the same spectral profiles of the emission and excitation spectra (Fig. 2(a,b), ESI Figs S6 and S7) between the as-prepared Ln-MOFs are consistent with no structural alterations or significant defects caused by interactions between Ln-MOF nanocrystals and the components of the cell wall. In particular, no substantial changes have been observed in the lifetime of the excited state (τ), quantum efficiency (η) and intensity ratio I(^5^D_0_ → ^7^F_2_)/I(^5^D_0_ → ^7^F_1_) data of fungi/Eu-MOF derivatives (ESI Table S1, Figs S8–S10). This suggests that cell wall components do not affect the luminescence mechanism of the Ln^3+^ ions in the biohybrids^37,38^. Additionally, the maintenance of the spectral profile of the excitation for all biohybrids (Figs S6 and S7) corroborates with the hypothesis of the absence of a cooperative interaction between the fungal cell wall and Ln-MOF microcrystals, in the dynamics of the luminescence process involving the electronic states of the mellitate ligand and Ln^3+^ íons^37,38,40^.

**Figure 3:**
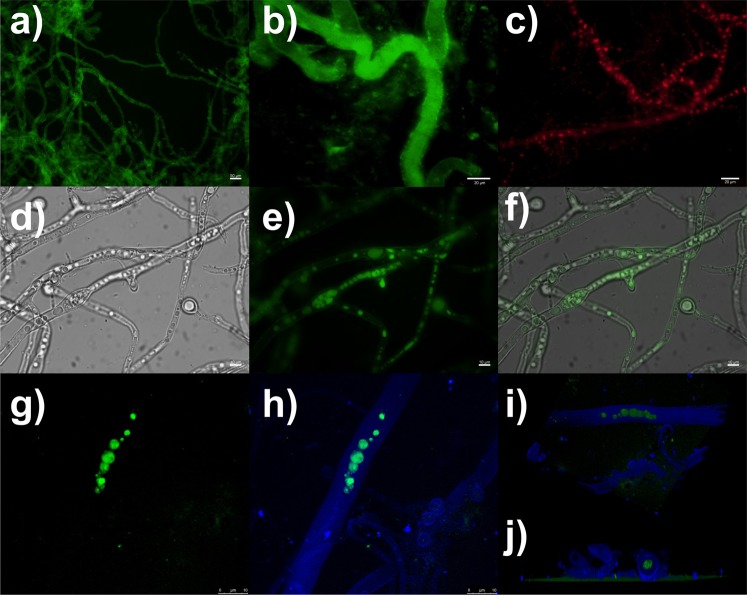
Fungal species labelled with Ln-MOFs. Fluorescence images of (**a**,**b**) *P*. *macrosporus*/Tb-MOF and (**c**) *Trichoderma sp*./Eu-MOF. Vacuolar system of the *Trichoderma sp*./Tb-MOF biohybrids in (**d**) bright field, (**e**) fluorescence and (**f**) composite images. The false colours are red and green for Eu-MOF and Tb-MOF, respectively. Confocal scanning laser images of *A*. *niger*/Tb-MOF: (**g**) fluorescently labelled organelles and (**h**) processed image emphasising the colocalisation of the organelles in the hyphal compartment. 3D cell reconstruction of CLSM images in a (**i**) longitudinal view and an (**j**) axial view, highlighting the inner localisation of the luminescent organelles in the hyphae.

Fluorescent imaging techniques present inherent advantages, such as sensitivity, simplicity and fast response, for studying events at the cellular and subcellular levels. However, traditional organic dye probes, such as green protein fluorescent and small organic dyes, present some limitations, including low photostability, low signal-to-noise ratio, phototoxicity and hydrophobicity^41^. Alternatively, inorganic quantum dots require a laborious and time-consuming step of surface functionalisation to become biocompatible^42^. The Ln-MOFs studied in this work possess excellent properties that can be useful for *in vivo* fungal images as they do not require any surface modification to be biologically compatible and possess intense photoemission that permits easy discrimination between the target and the background emissions. Biohybrid fungi/Ln-MOFs were examined under a conventional fluorescent microscope to display their luminescent properties. Figure 3 shows representative green and red emissions for *P*. *macrosporus*/Tb-MOF and Eu-MOF, respectively. In Fig. 3(a,b), luminescence is undoubtedly observable, due to the thick layer of green emitter attached to the fungal surface.

Although both Ln-MOFs present similar emission intensities for dried biohybrids, we observed a low intensity of the red light when analysing living fungi/Eu-MOF investigated under the microscope. This observation results from the efficient non-radiative deactivation of luminescence through coupling between the hydroxyl group of water (O-H strength) and the f electronic levels of the Eu^3+^ ions^23^. Therefore, despite the presence of chromophore species deposited on the fungal surface, the photoluminescence signal is quite low for living fungus/Eu-MOFs in aqueous environments.

Considering the ability of filamentous fungi in accumulating different particulate materials over the cell wall^32–34,43,44^, we also hypothesised whether these microorganisms would be able to internalise luminescent particles and store them in the cytosol or bind them to specific cellular components. Initially, we were intrigued by some results of fluorescent microscopy of the fungi/Eu-MOF, where it was possible to observe bright red spheres located along the hyphae (see Fig. 3(c)). For example, despite the difficulty in observing a luminous signal at the outer surface of the *A*. *niger* /Eu-MOF hypha (see ESI, Fig. S12), it was relatively easy to observe these bright spheres that followed the tubular structure of the hyphae. We concluded, therefore, that Ln-MOFs could concentrate at organellar lumen in the inner fungal cells, evidencing fluorescence even for Eu-MOF.

By selecting arbitrary regions at the periphery of the fungal biohybrid mycelia, the uptake of Tb-MOF improved the visualisation of spherical and tubular organelles within cells (Fig. 3(d,f)). These experimental findings confirm that Ln-MOFs play a role as photoluminescent probes, which were concentrated, apparently, at the fungal vacuoles of *Trichoderma* sp. biohybrids (see Figs S11–13 for other examples). Vacuoles are large organelles with multiple cellular functions, including reservoir and decontaminating the cytoplasm by sequestrating toxic substances^45^. To verify the ability of microorganisms in the internalisation of Ln-MOFs instead of luminescent Tb^3+^ or Eu^3+^ ions, fungal cultures were grown under the same conditions, but by adding Ln(NO_3_)_3_ (Ln = Eu or Tb) during cultivation. We observed growth inhibition for all species studied in this work, because lanthanides ions are not essential nutrients and, apparently, the Ln(NO_3_)_3_ is a poor source of nitrogen for the fungi. The microorganisms cultivated in the presence of Ln^3+^ ions revealed only fungal intrinsic fluorescence (see ESI, Fig. S14 for Tb(NO_3_)_3_).

In order to confirm that the fluorescent signal arises from internalised Ln-MOFs stored in the fungal organelles, we then performed additional experiments using confocal scanning laser microscopy (CSLM, Fig. 3(g–j)). Upon excitation at 405 nm, the Tb- and Eu-MOFs showed emission spectra that coincide with the data obtained by using UV light as the excitation source (ESI Fig. S15). Although the steady-state emission of the biohybrids displays co-fluorescence of fungal hyphae and Ln-MOFs (Fig. S16), the time-resolved emissions are composed of only f-f transitions. Since UV light is known to be prejudicial to the microorganisms, we substitute the excitation source in our experiments by using a low-energy microscope with a wavelength at 405 nm to study these biosystems. Specifically, Fig. 3(g) emphases the fluorescent vacuoles of the *A*. *niger*/Tb-MOF while Fig. 3(h) shows a representative processed image also taking into account the fungal autofluorescence. Thereby, based on analysis of 3D cell reconstruction (Fig. 3(h–j)), it is possible to confirm that the fluorescence emission of Ln-MOFs originates from organelles within the hyphal compartment. Up to now, the exact nature of the internalisation mechanism of nanosized materials by fungi is currently an unknown process^8,46,47^. In one of few studies regarding fungal uptake of nanoparticles, Whiteside *et al*. speculated that glycine, arginine or chitosan conjugated CdSe/ZnS quantum dots can incidentally be acquired by *Penicillium solitum* fungus through biological transporters for amino acids, peptides, proteins and polysaccharides^46^. Our next step is to better understand the assimilation of Ln-MOFs by microorganisms, particularly, whether an enzymatic breakdown of crystals near the hyphal body could facilitate the uptake of small particles by fungi.

The affinity of microorganisms to the crystals of Eu or Tb-MOF was evaluated by growing the fungal cultures in a media with an equimolar mixture of Eu-MOF and Tb-MOF during cultivation. XRD patterns (showed in ESI Fig. S17) display peaks indexed to a physical mixture of Ln-MOFs, indicating the integration of both Tb and Eu-MOFs at fungal hyphae. In line with this, the emission spectra of *P*. *macrosporus*/EuTb-MOFs and *Trichoderma* sp./EuTb-MOF biohybrids (ESI, Fig. S18(a)) exhibited a spectral profile composed of both Eu and Tb emission (^5^D_0_ → ^7^F_J_ and ^5^D_4_ → ^7^F_J’_; J = 0, 1, 2, 3 and 4, and J’ = 6, 5, 4, 3, 2, 1 and 0), corroborating the incorporation of Eu- and Tb-MOFs at the fungal hyphae. Moreover, the colour coordinate of photoluminescence exhibited by *P*. *macrosporus*/EuTb-MOFs and *Trichoderma* sp./EuTb-MOFs biohybrids are very close, (0.368; 0.539) and (0,369; 0,539), respectively (ESI, Fig. S18(b)). Owing to the energy transfer pathways between Eu (acceptor) and Tb (donor) centres being blocked in the physical mixture of the adjacent Eu- and Tb-MOFs particles, the spectral profile (relative emission band amplitude or colour coordinate) can be used to estimate the relative molar ration between Ln-MOFs^48–50^. Thus, we can infer that the amounts of Eu- and Tb-MOFs incorporated by the *P*. *macrosporus* and *Trichoderma* sp. are quite similar, as expected (see ESI, Fig. S18(c,d)). To corroborate this assumption, we carried out a control experiment mixing Eu and Tb-MOFs at molar ratios (Eu-MOF:Tb-MOF) of 1:9, 1:3, 1:1 and 3:1. According to the emission spectral profile exhibited by the solid physical mixture of Ln-MOFs (see Fig. S19 (a) in ESI), we can notice that the maximum intensity of the ^5^D_0_ → ^7^F_2_ (Eu^3+^) and ^5^D_4_ → ^7^F_5_ (Tb^3+^) transitions changed with the molar ratio, in which they are equal at the molar ratio of 1:1, which confirms that fungi assimilated the same amount of Eu and Tb MOFs. This evidence is clarified through the observation of a linear relationship between the intensity ratios of the transitions ^5^D_0_ → ^7^F_2_ (Eu^3+^)/^5^D_4_ → ^7^F_5_ (Tb^3+^) and the molar ratio n(Eu-MOFs)/n(Tb-MOFs) in the Ln-MOFs physical mixture (ESI, Fig. S19(b)).

**Figure 4:**
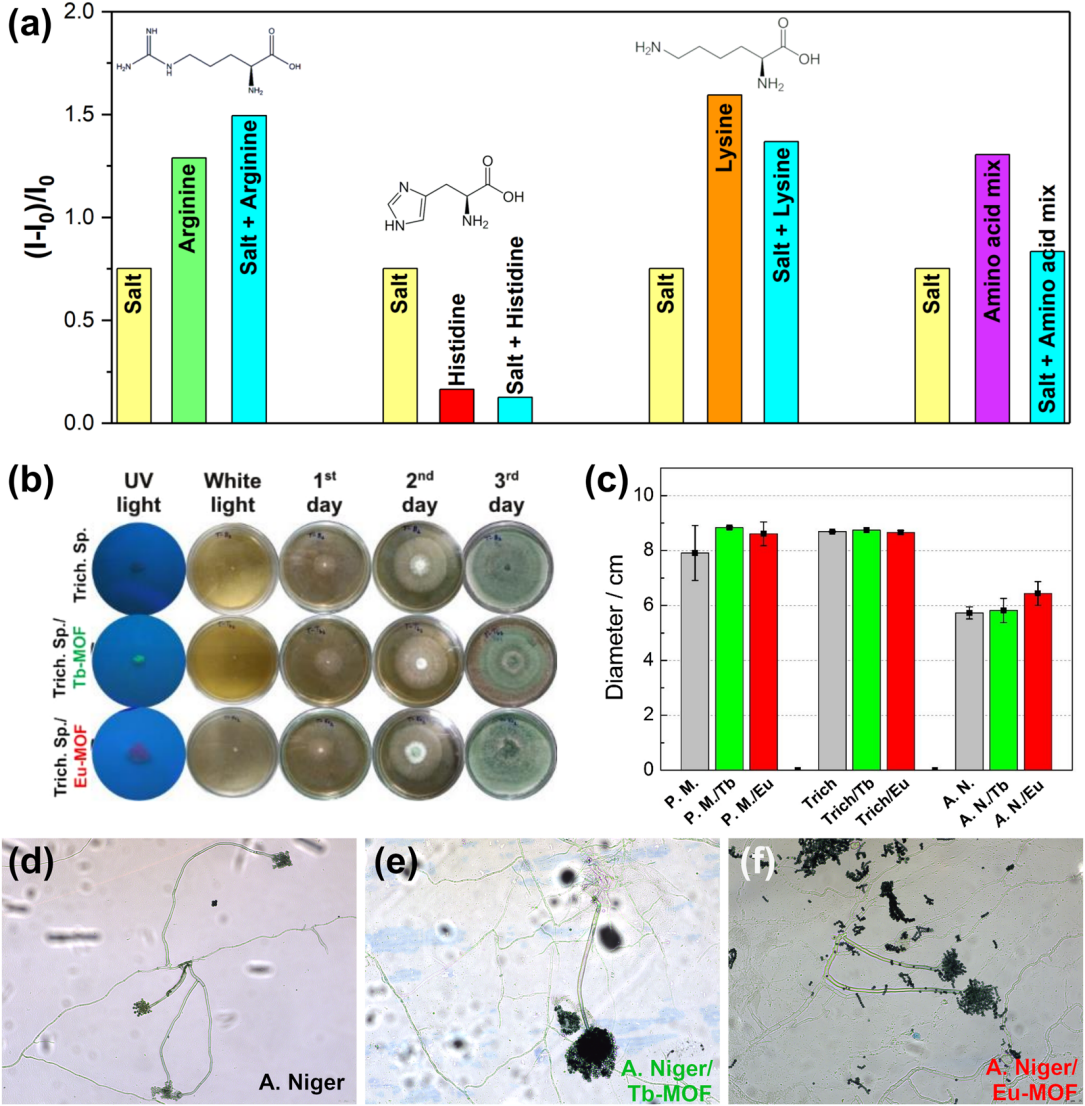
(**a**) Relative enhancement in the integrated emission intensity (from 478 to 600 nm) of Tb-MOF in the presence of salts and amino acids. I_0_ and I are the integrated emission intensities of the aqueous suspension of Tb-MOF without and with salts and/or amino acids, respectively. (**b**) Digital image of the diameter of *Trichoderma* sp. and *Trichoderma* sp. biohybrid colonies after three days of growing in Petri dishes. Note that the development of the colonies was similar for the three samples. The images obtained under ultraviolet light were enlarged for greater clarity. (**c**) Comparative radial growth after seven days of cultivation of *P*. *macrosporus*, *Trichoderma* sp. and *A*. *niger*. and their respective Eu and Tb-MOFs biohybrids. Filamentous cells grown from the native (**d**) *A*. *niger* fungus and its (**e**) *A*. *niger*/Tb-MOF and (**f**) *A*. *niger*/Eu-MOF biohybrids, respectively.

Moreover, we observed in the images of fluorescence microscopy that the luminescence intensity of Tb-MOF-based biohybrids is more intense than those of Eu-MOF-based biohybrids. Thus, to understand this behaviour, we perform one additional experiment, acquiring the emission spectra of the Ln-MOFs in a simulated vacuolar medium. Within this line, to evaluate the high luminescence intensity of Ln-MOFs inside of fungal vacuoles and whether it is correlated to a concentration effect, we evaluate the luminescence properties of MOFs in aqueous suspensions in the presence of some metal salts (MgCl_2_, KH_2_PO_4_ and NaCl) and amino acids (arginine, histidine and lysine), according to the composition of a typical extract of isolated fungal vacuoles^51^. Remarkably, the metal salts and amino acids induce a significantly increased luminescence of the Tb-MOF, mainly for arginine and lysine, in which an enhancement above 150% is displayed (see histogram displayed in Fig. 4(a)). The weak increase caused by the histidine is correlated to an inner filter effect, due to absorption of this amino acid in the region of the excitation source, 312 nm (see Fig. S20)^52,53^. Due to the maintenance of the spectral profile of the Tb-MOF (see Fig. S21), we assign this luminescence enhancement to coordination and supramolecular interactions, respectively, of the metal ions and amino acids to mellitate ligands at the surface of the MOF nanoparticles^54–57^. These interactions block the surface luminescence quenching, commonly verified in luminescent nanoparticles^58,59^. In contrast, there is no change in the emission intensity of the Eu-MOF (see Fig. S22 in ESI). In spite of this, we see an intense red-light emission from the fungal vacuoles (see for instance Fig. S12 in ESI). The reason for this is not clear yet. One point to be highlighted is that the intravacuolar composition of the fungi is much more complex than the simulated composition of vacuoles in our experiments. Therefore, a possible explanation would be the existence of unknown biomolecules and ions inside the vacuoles which, when interacting on the surface with the mellitate ligands of the Eu-MOFs nanoparticles, could avoid the quenching effect due to the O-H oscillator strength of the coordinated water molecules at the Eu^3+^ ions, as aforementioned.

The biotoxicity of Ln-MOFs on fungal species was evaluated based on the analysis of cell survival after assimilation of the abiotic components by these microorganisms. Initially, we executed the alamarBlue test, which involves the detection of metabolic activity through the assimilation of a redox indicator that changes its colour from blue (oxidised form) to pink (reduced form) during the cell growth. AlamarBlue is widely used to detect quantitatively the proliferation of eukaryotic cell lines (human and animal), bacteria and fungi^55,56^. For instance, Santos and co-workers reported cellular activity using an alamarBlue assay of spherical pellets of *Aspergillus fumigatus* after experiments of electrochemical degradation of hydrogen peroxide using integrated microelectrode in the mycelium clumps^57^. Although we observed changes in absorbance values, indicating cell viability of fungal species and their biohybrids (pink color), it was not possible to obtain quantitative measurements since those fungi were grown in submerged static cultures, forming a dispersed micellar network. Thus, it is not possible to standardise the amount of mycelium in the replicates, leading to large errors in the absorbance values.

An additional approach was also carried out by evaluating the total radial growth of microorganisms on a solid surface containing an agar-dextrose medium and their phenotypic characteristics after Ln-MOF deposition. In this case, we assumed that the direct evidence of an inhibitory effect from Tb- and Eu-MOFs over microorganisms would be the absence of their mycelial growth. For this experiment, small fragments of each mycelium were inoculated in Petri dishes containing a mixture of agar and 1.0% dextrose (w/v). The native fungi and the biohybrids were cultivated under the same environmental conditions, and the radii of colonies were daily measured using a caliper (see Fig. 4(b) for growth profile of the fungus *Trichoderma sp*. and its biohybrids for three days). Figure 4(c) shows the diameter of colonies after seven days of cultivation for the all biosystems studied in this work. As can be observed, the profiles of fungal growth are quite similar for the biohybrids and native fungi, evidencing similar radial growth rates in all phases of the growth curve. We also examined the aspects of the macroscopic appearance of the colonies, such as colour, texture and the production of pigments. These observations have shown that there were no significant differences between the colony growth by native fungi and their biohybrid counterparts, suggesting that Ln-MOFs would not produce any permanent modification in the fungal structures.

To complement the biotoxicity investigation, the micromorphology of the filamentous cells originating from native fungi and the biohybrid counterparts were compared using Riddell’s slide cultures technique^60^. Succinctly, mycelial fragments of each species and their biohybrids were inoculated in a sterile potato dextrose agar (PDA) placed on a sterile microscopy slide. Subsequently, the mycelium-PDA set was protected with a coverslip, and the whole set was incubated in a Petri dish for seven days. For comparison purposes, careful observation of morphological characteristics of hyphal cells was carried out. As a result, we determine that there is no difference between cells originating from native fungi and fungi/Ln-MOF biohybrids (see Fig. 4(d,f) for filamentous cells growth from native *A*. *niger* and their biohybrids). Thus, we conclude that the presence of Ln-MOFs on the fungal ultrastructure does not bring apparent toxic effects to the microorganisms being. Therefore, this methodology is promising for the bioimaging of fungi.

## Conclusions

This work has demonstrated that controlled deposition of Ln-MOFs on filamentous fungi converts native microorganisms into photoluminescent living entities. We have confirmed the integration of Ln-MOFs onto microorganisms through SEM, XRD and fluorescence spectroscopy. Tb- and Eu-MOFs form a fluorescent carapace over the cell and, most importantly, tiny Ln-MOFs particles were internalised in specific cellular components, making this strategy relevant for living imaging analysis. The integration of Ln-MOF nanoparticles to the fungal structure does not present any apparent damage to the vital functions of the microorganisms, whose development is similar to that of native cells. Control experiments simulating components found in the vacuolar medium revealed that the internalisation of Ln-MOFs could intensify the luminescence process, especially for the Tb-MOF, facilitating the visualisation of the organelles. To conclude, we believe that our findings may be relevant for research involved with the treatment of fungal infections. Fungal diseases are difficult to treat since their rigid cell wall presents a barrier to drug penetration. Eventually, the uptake of antifungal agents encapsulated in the MOF cavities by the microorganisms would be a promising strategy in the treatment of such infections^61^.

## Methods

### Chemicals

Mellitic acid and lanthanide oxides (99.99%, where Ln^3+^ = Eu^3+^ or Tb^3+^) were purchased from Aldrich and used without further purification. The lanthanide salts (Ln(NO_3_)_3_.5H_2_O) were obtained by reaction of nitric acid with the corresponding lanthanide oxide at 60–80 °C. The pH was adjusted to 6.0 by water vaporisation and the solid salts were collected by vacuum drying at 60 °C.

### Synthesis of Ln-MOF (Ln = Eu or Tb)

Lanthanide metal-organic frameworks (Ln-MOFs or [Ln_2_(Mell)·6H_2_O]) were synthesised by adding 171 mg of mellitic acid (0.5 mmol) and 223 mg of lanthanide salt (0.5 mmol) into 5.0 mL of deionised water under constant magnetic stirring for 5 min. Then, 1.5 mL of ethanol (30% in volume) was slowly added to the solution maintained at rest to induce rapid crystal nucleation. White microcrystals were obtained in a yield of 53%.

These crystals were submitted to high intensity ultrasound irradiation (Vibracell VC 130, Sonics Instruments) using a titanium tip (0.6 cm in diameter) immersed approximately 2.0 cm in the liquid to reach smaller particles sizes. For this purpose, 0.06 g of Ln-MOFs were sonicated in 50 mL of deionised sterile water for 2 h, passed through a syringe filter (pore size of 0.8 µm) and used immediately for microorganism cultivation.

### Preparation of biological samples

The fungi *Phialomyces macrosporus* and *Trichoderma* sp. were isolated from dead leaves from plants of the Environmental Protection Area of Lagoa e Dunas do Abaeté, Salvador, Bahia, Brazil, and deposited in the culture collection of the Laboratory of Biotechnology and Chemistry of Microorganisms (LBQM - UFBA). *Aspergillus niger* (ATCC-16404) was acquired from Fundação André Tosello, Campinas, São Paulo, Brazil.

These fungi were subcultured from their stock water flasks (Castellani method) on potato dextrose agar and the inoculum suspensions were prepared from the fresh cultures (seven-day old) by rubbing the colonies with a sterile loop and transferred to a sterile tube that was shaken vigorously with a Vortex mixer. The inoculum size was determined by microscopic enumeration with a cell-counting hematocytometer (Neubauer chamber), where the *Trichoderma* sp. spore suspension was 1.16 × 10^7^ spores/mL, *Phialomyces macrosporus* was 3.75 × 10^4^ spores/mL, and *Aspergillus niger* exhibited 9.50 × 10^5^ spores/mL.

Fungi/Ln-MOF biohybrids were cultivated through the following protocol. In a penicillin flask was added 4.0 mL of deionised sterile water, 5.0 mL of a solution containing sonicated Ln-MOFs (1.2 mg/mL) and 1.0 mL of a sterile solution containing 2.0% glucose. Then, 100 µL of a spore suspension were added to the growing medium and allowed to grow at 25–28 °C for 120 h in the dark. Experiments were done in triplicate. After cultivation, the mycelial mass of fungi/Ln-MOF biohybrids were copiously rinsed with deionised water to remove any weakly bonded particulate Ln-MOFs. For XRD, SEM and photoluminescence measurements, the biohybrids were dried using critical point drying, as described in the literature^35^. Fluorescence microscopy analyses were carried out in small fragments of mycelium stored in deionised water without any other treatment.

### Preparation of fungi/Ln-MOF biohybrid samples in Petri dishes

To evaluate the fungi/Ln-MOF biohybrid viability, the radial growth of these samples was monitored. Sterile Petri dishes were prepared with ~20 mL of the culture medium of dextrose (20 g.L^−1^) and agar (15 g.L^−1^), and kept in a sterile laminar flow chamber under UV light until culture media solidification. A small fragment of fungi/Ln-MOF biohybrids and native-type ones were transferred to test Petri dishes and placed in the centre. The dishes were incubated at 25–28 °C for 168 h. The culture radial diameters were measured along four axes. Each evaluation was made every 12 h in the first two days, and every 24 h from the third day. All experiments were performed in triplicate. The statistical analysis of the mean and the standard deviation of the triplicates was achieved using Student’s t-test at the 0.05 level with a confidence interval of 95%.

### General instrumentation

SEM images were acquired on a JEOL JSM5400 (LAMUME, IF-UFBA). Energy-dispersive X-ray (EDS) spectra were obtained using a SEM microscope by selecting regions with 500× magnification. The X-ray diffraction (XRD) patterns were performed on a Bruker D8 Advanced diffractometer over a 2θ range from 5° to 50°, a velocity of 5°/min and increments of 0.02°. Simulated powder X-ray diffraction patterns (PXRD) were measured through the single crystal X-ray diffraction data (CIF file) as an input and the Mercury 3.8 software. Particle size distribution and zeta potential were obtained in a Zetatrac Legacy equipment (at CETENE – Centro de Tecnologias Estratégicas do Nordeste, Recife – PE). Photoluminescence measurements were carried out on a spectrofluorimeter HORIBA-JOBIN YVON FLUOROLOG-3 equipped with a continuous 450 W xenon lamp and UV xenon flash tube for excitation. All emission spectra were corrected by spectral response of the monochromators using a silicon photodiode reference detector, to monitor and compensate for variation in the xenon lamp output, using typical correction spectra provided by the manufacturer. For fluorescence microscopy, fragments of fungal mycelium were copiously rinsed with deionised water and held between a coverslip and a microscope glass slide without any other treatment. The samples were viewed using an Olympus BX51 microscope equipped with a 100× magnification oil-immersion objective and a Retiga 2000R monochrome CCD camera. Samples were illuminated with a 100 W Hg vapor source using a BP380-385 excitation filter and a BA420 emission filter. The images obtained were processed using a Leica LAS X software. Confocal scanning laser microscopy analyses were performed using a Leica TCS SP8 microscope, equipped with a 63× magnification objective and a laser source at 405 nm. The luminescence properties of aqueous suspensions of Ln-MOFs influenced by salts and amino acids were studied as follows: 300 µL of a sonicated aqueous suspension of Eu or Tb-MOF (2.4 mg/mL) were added to a 3.5 mL quartz cuvette containing deionised water and a mixture of salts (MgCl_2_ (2.7 mM), KH_2_PO_4_ (0.7 mM) and NaCl (1.5 mM)) and/or amino acids arginine (13.6 mM), histidine (1.9 mM) and lysine (1.8 mM). Luminescence analyses were performed using a Shimadzu RF-5301PC spectrofluorophotometer equipped with a continuous 450 W xenon lamp. For morphological analyses, we used the well-established Riddell methodology for the preparation of coverslips containing cells grown from native fungi and their respective biohybrid. The samples were investigated in an inverted Leica DMi8 microscope at Gonçalo Moniz Research Center - Fiocruz/BA.

## Supplementary information

ESI
