# Supplementary material for: Photoluminescent organisms: how to make fungi glow through biointegration with lanthanide metal-organic frameworks
Source: Sci Rep. 2019 May 13;9:7302. doi: 10.1038/s41598-019-43835-x (PMC6513872; doi:10.1038/s41598-019-43835-x)
Supplement: Supplementary file 1 — ESI [file 41598_2019_43835_MOESM1_ESM.docx]

Supporting Information

Photoluminescent organisms: how to make fungi glow through the biointegration with lanthanide metal-organic frameworks

Jeferson Rosário, Leonis L. da Luz, Regina Geris, Jéssica G. S. Ramalho, Antônio F. da Silva, Severino Alves Júnior* and Marcos Malta*

**Results**


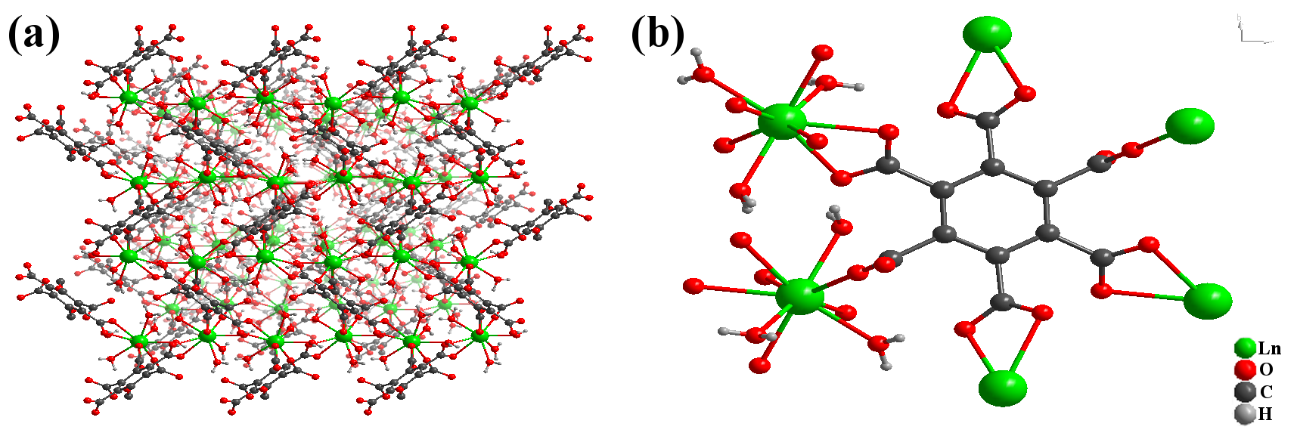


**Figure S1:** Coordination network of Ln-MOF viewed along c axis (a) and, coordination environments of mellitate and Ln^3+^ ions in the Ln-MOF network (b). (Rosário, J.; da Luz, L.L. *et al.*)


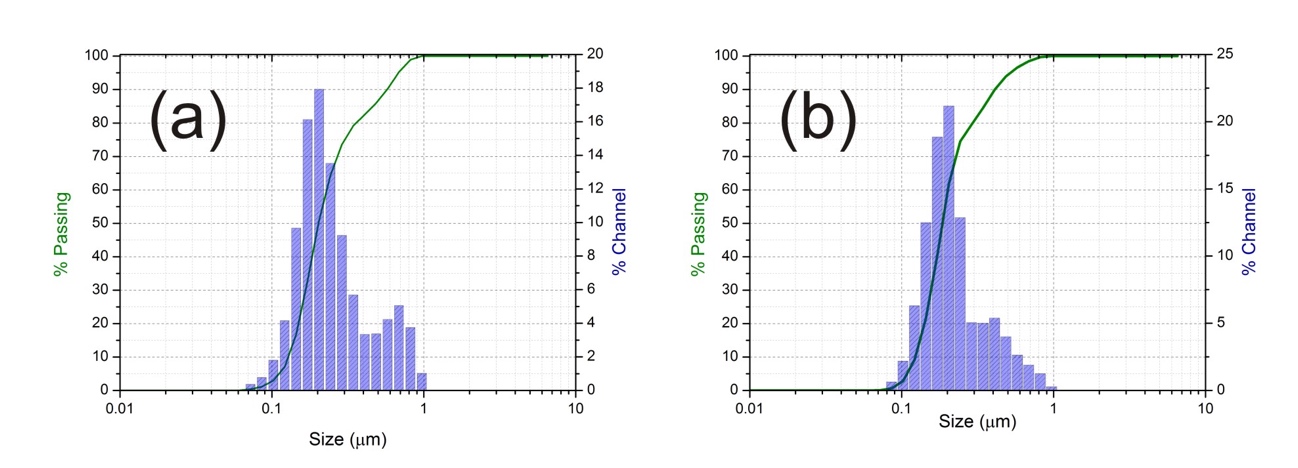


**Figure S2:** Particle size distributions for (a) Tb-MOF and (b) Eu-MOF after treatment with high intense ultrasound. (Rosário, J.; da Luz, L.L. *et al.*)


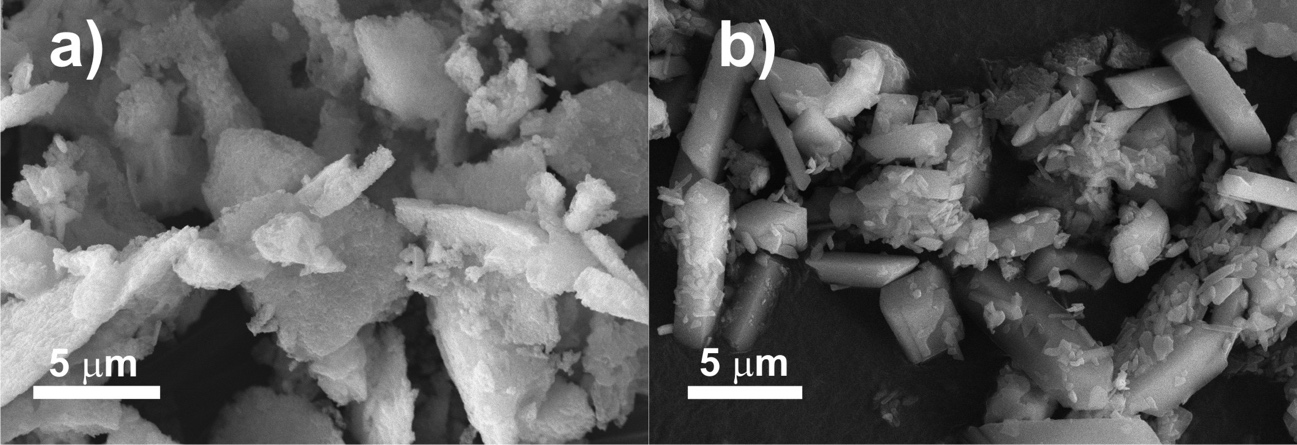


**Figure S3:** Secondary electrons mode of SEM images of a) Tb-MOF and Eu-MOF after treatment with high-intensity ultrasound. It can be observed large Ln-MOFs aggregates due to the solvent evaporation. (Rosário, J.; da Luz, L.L. *et al.*)


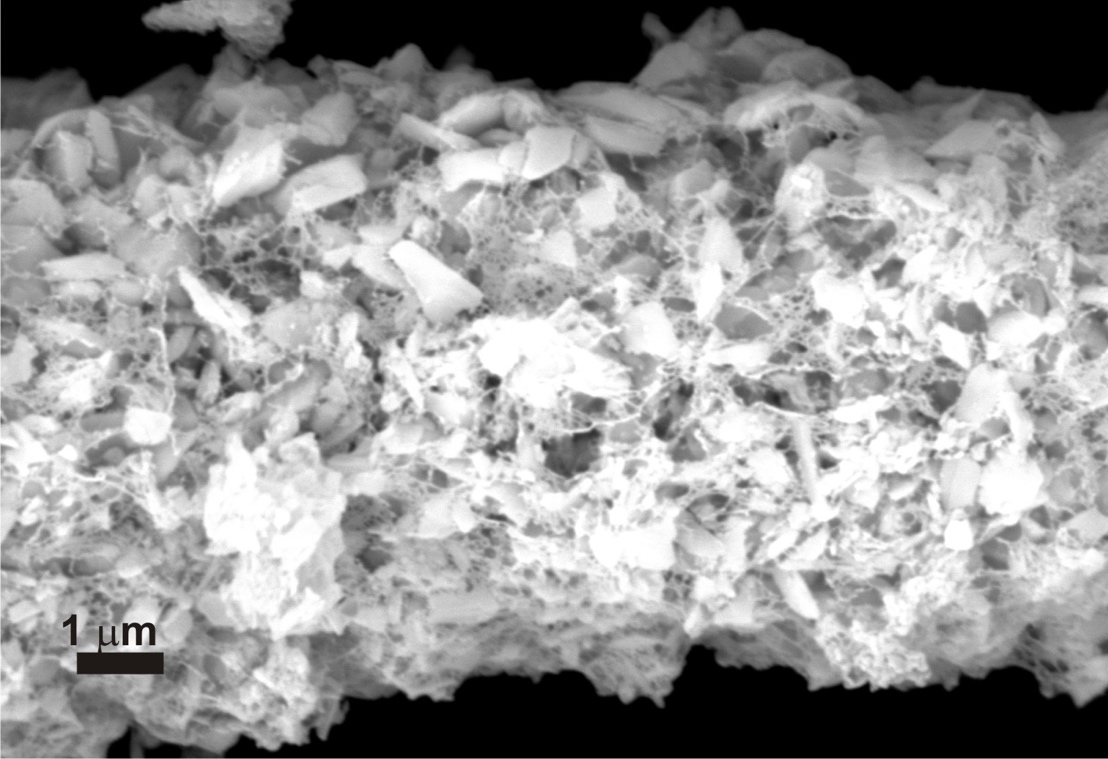


**Figure S4:** Backscattering mode of SEM image of an isolated hyphae of *P. macrosporus*/Eu-MOF. Note particulate abiological material entrapped into biopolymeric network of the fungal wall. (Rosário, J.; da Luz, L.L. *et al.*)


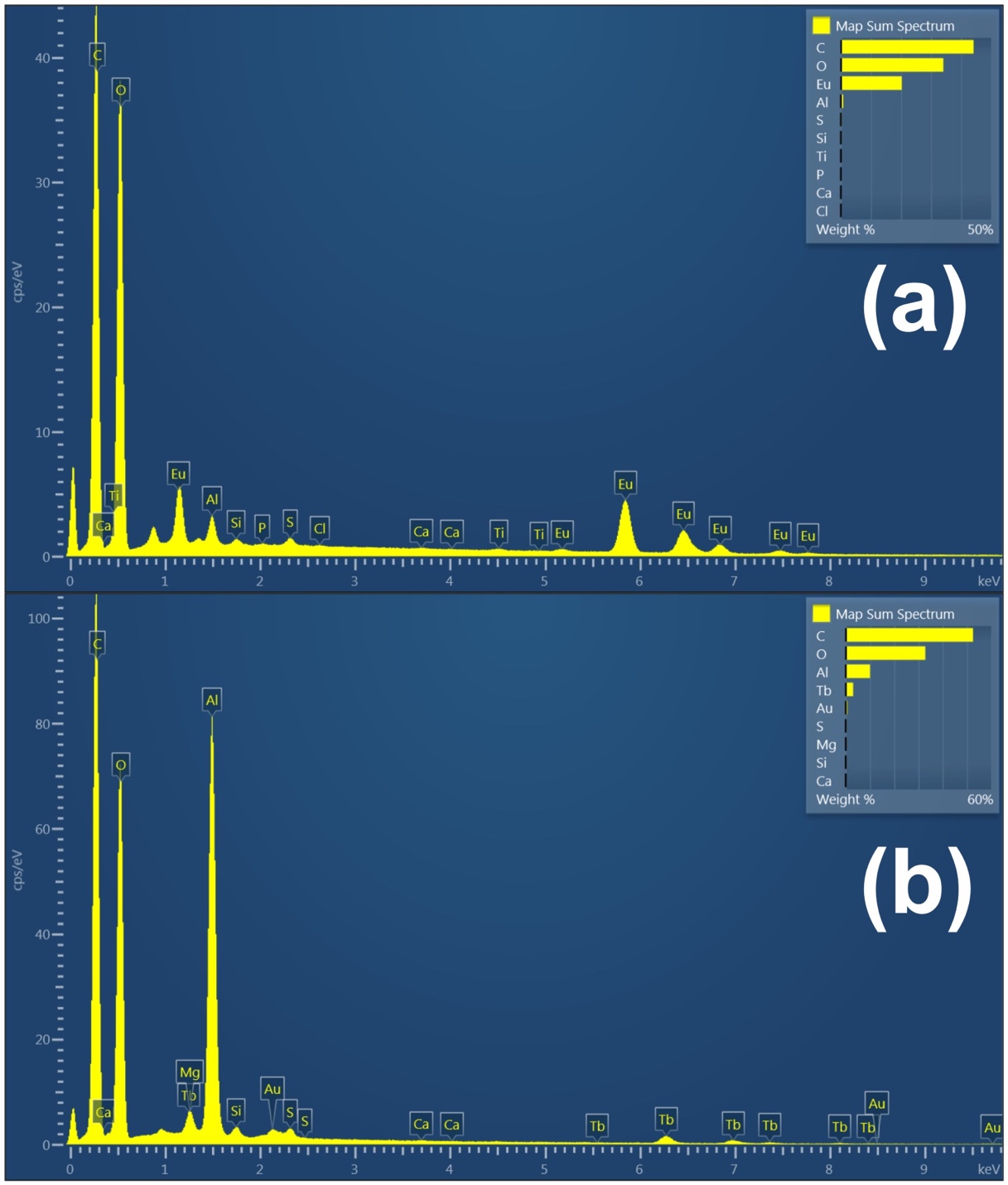


**Figure S5:** EDX analyses of (a) *P. macrosporus*/Eu-MOF and (b) *P. macrosporus*/Tb-MOF. (Rosário, J.; da Luz, L.L. *et al.*)

The experimental emission quantum efficiency (*η*), meaning, direct lanthanide-excitation-based efficiency, were determined by the experimental values of the lifetime of the ^5^D_0_ emitting level (*τ*), probabilities for the radiative (A_rad_) and total (A_Total_) decay processes, respectively, according to the following equations. The emission quantum efficiency (or intrinsic quantum yield) was determined by equation:^[1, 2]^

| $\eta= \frac{A_{rad}}{A_{rad}+A_{nrad}}$ | (1) |
| --- | --- |

The spontaneous emission probability (A_0-J_), or Einstein’s coefficient, of the transitions from ^5^D_0_ emitting level to ^7^F_J_ levels, arising from Eu(III) ion was calculated, based on the emission spectrum, by the follow equation: ^[1,2]^

| $A_{0\to J}= \frac{\upsilon_{0\to1}}{S_{0\to1}}\frac{S_{0\to J}}{\upsilon_{0\to J}}A_{0\to1}$ | (2) |
| --- | --- |

Where 𝜐_0-1_ and 𝜐 _0-J_ correspond to the energy barycenter of the ^5^D_0_→^7^F_1_ and ^5^D_0_→^7^F_J_ transitions (in cm^-1^), respectively. In the similar way, S_0-1_ and S_0-J_ are the integrated intensity of the emission bands corresponding to the ^5^D_0_→^7^F_1_ and ^5^D_0_→^7^F_J_ transitions, respectively. Considering that A_0-1_ rate is almost insensitive to changes in the chemical environment around the europium ion, the value of A_0-1_≈ 50 s^-1^ is taken and we assuming that the refraction index (n) for the samples in solid state equal to 1.5.^[2]^ The total decay rate is defined by A_tot_ = (1/*τ*) = A_rad_ + A_nrad_. Thus, based on the experimental lifetime (*τ*) and the A_rad_ rate, it possible to determine the nonradiative rate (A_nrad_).

Luminescence decay curves, to an average of three intensity measurements performed automatically by the equipment, of the Ln-MOFs and Fungi/Ln-MOFs materials were fitted by the equation I(t) = I(0) exp(-t/τ) using a curve-fitting program. It indicates the presence of only one site of symmetry around Ln(III) ion.

**Table S1:** Radiative (A_rad_) and nonradiative (A_nrad_) decaying rates, emission life-time of europium emission at λ_Ex_ = 312 nm (τ_obs_), quantum efficiency (η) and intensity ratio (I(^5^D_0_→^7^F_2_)/I(^5^D_0_→^7^F_1_)).

| Material | A_rad_(s^-1^) | A_nrad_(s^-1^) | τ_obs_(ms) | η (%) | I(^5^D_0_→^7^F_1_)/I(^5^D_0_→^7^F_1_) |
| --- | --- | --- | --- | --- | --- |
| Eu-MOF | 538,4 | 4724,8 | 0.19 | 10 | 7,7 |
| *A. niger*/Eu-MOF | 577,4 | 4978,1 | 0.18 | 10 | 8,3 |
| *Trichoderma* sp./Eu-MOF | 601,5 | 4398,5 | 0.20 | 12 | 8,3 |
| *P. macrosporus* /Eu-MOF | 582,2 | 4680,9 | 0.19 | 11 | 8,7 |


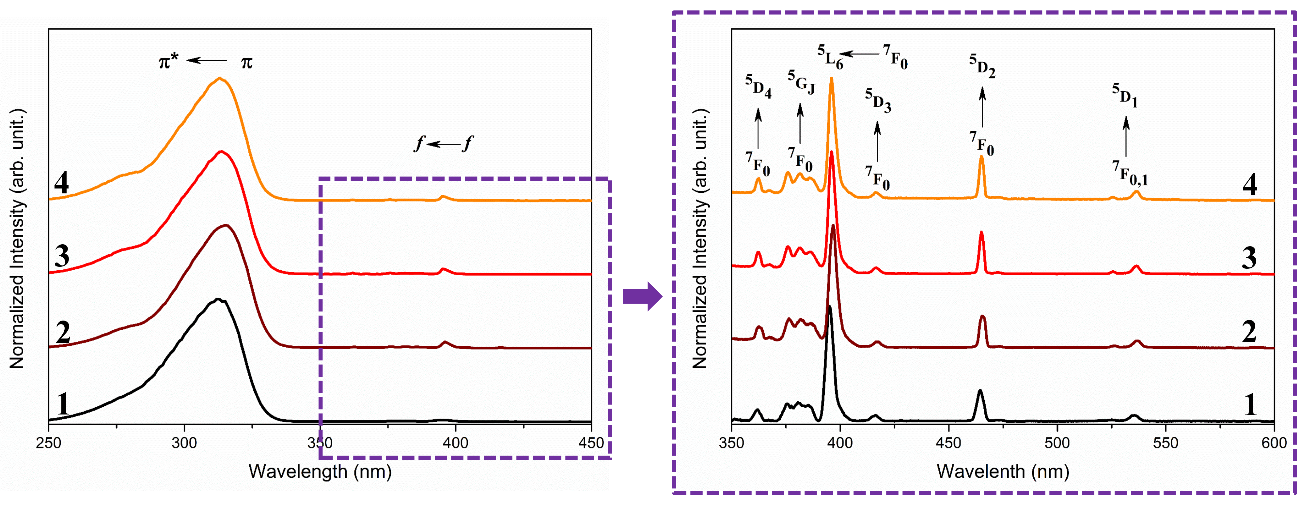


**Figure S6:** Excitation spectra of Eu-MOF (1), *A. niger*/Eu-MOF (2), *Trichoderma* sp./Eu-MOF (3) and *P. macrosporus*/Eu-MOF (4) obtained by monitoring emission at 615 nm (^5^D_0_→^7^F_2_). (Rosário, J.; da Luz, L.L. *et al.*)


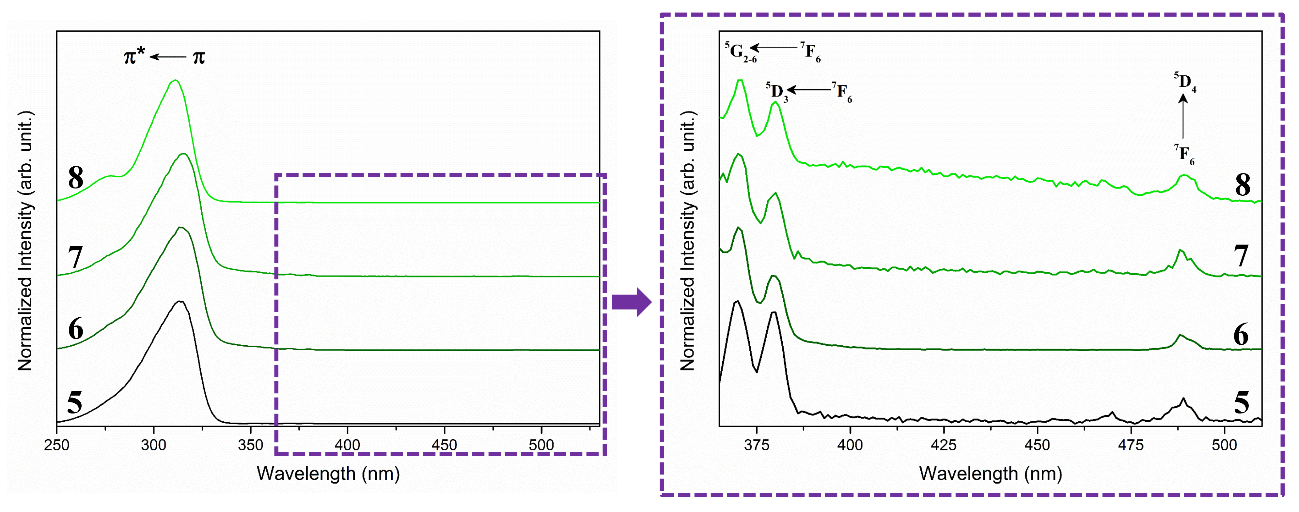


**Figure S7:** Excitation spectra of Tb-MOF (5), *A. niger*/Tb-MOF (6), *Trichoderma* sp./Tb-MOF (7) and *P. macrosporus*/Tb-MOF (8) obtained by monitoring emission at 545 nm (^5^D_4_→^7^F_5_). (Rosário, J.; da Luz, L.L. *et al.*)


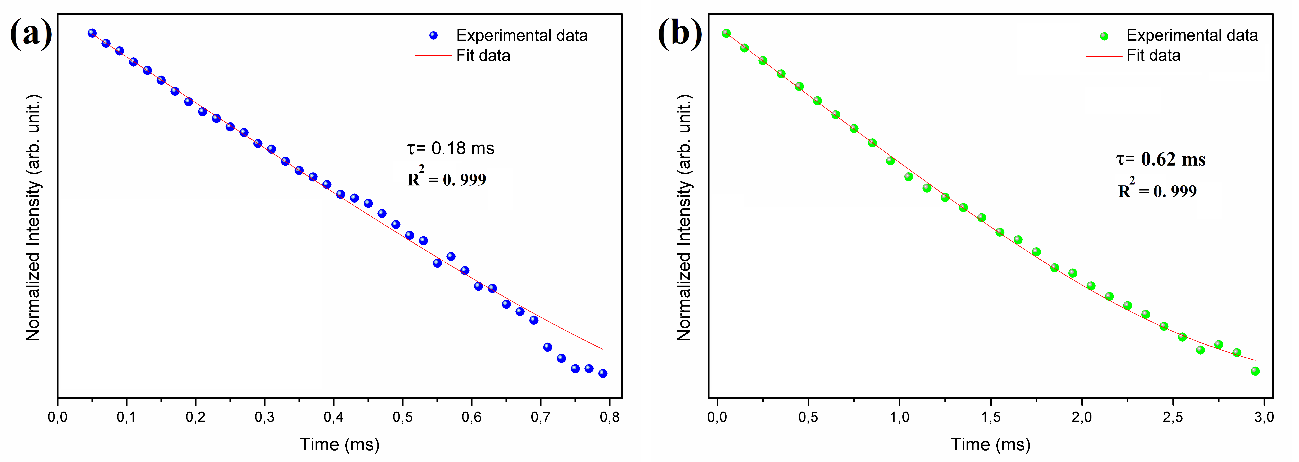


**Figure S8:** Normalized decay curves of Eu-MOF (a) and Tb-MOF (b) upon excitation at 312 nm and monitoring emission at 615 nm (^5^D_0_→^7^F_2_) and 545 nm (^5^D_4_→^7^F_5_), respectively. (Rosário, J.; da Luz, L.L. *et al.*)


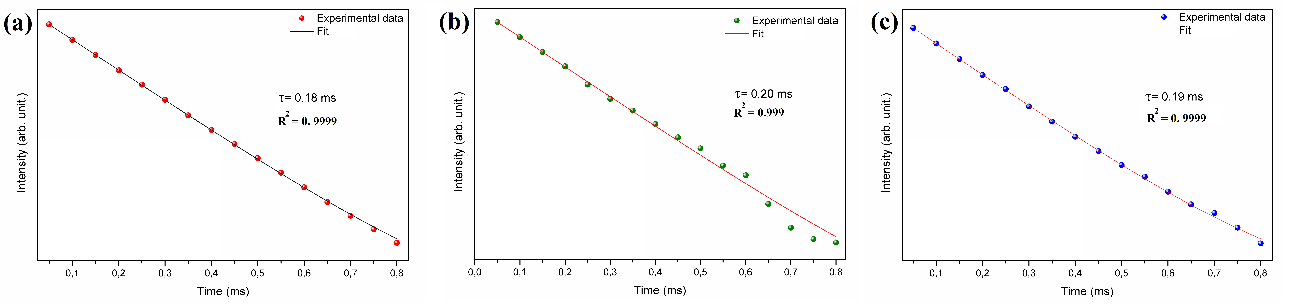


**Figure S9:** Normalized decay curves of *A. niger*/Eu-MOF (a), *Trichoderma* sp./EuMOF (b) and *P. macrosporus*/Eu-MOF (c) upon excitation at 312 nm and by monitoring emission at 615 nm (^5^D_0_→^7^F_2_). (Rosário, J.; da Luz, L.L. *et al.*)


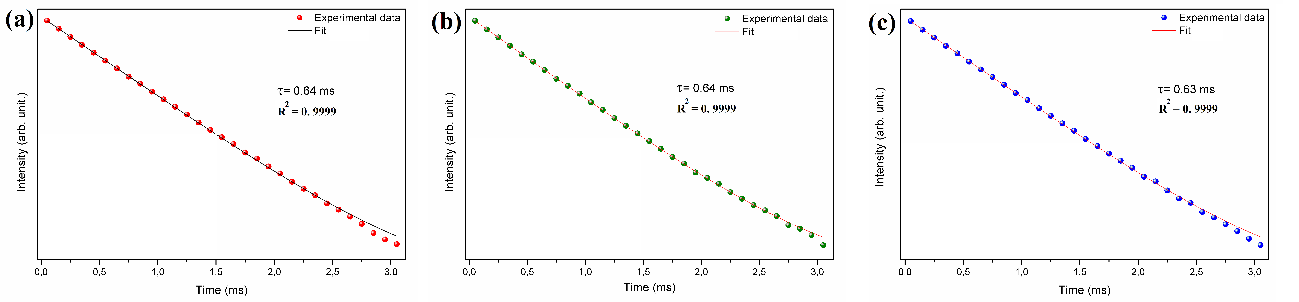


**Figure S10:** Normalized decay curves of *A. niger*/Tb-MOF (a), *Trichoderma* sp./Tb-MOF (b) and *P. macrosporus*/Tb-MOF (c) upon excitation at 312 nm and monitoring emission at 545 nm (^5^D_4_→^7^F_5_). (Rosário, J.; da Luz, L.L. *et al.*)


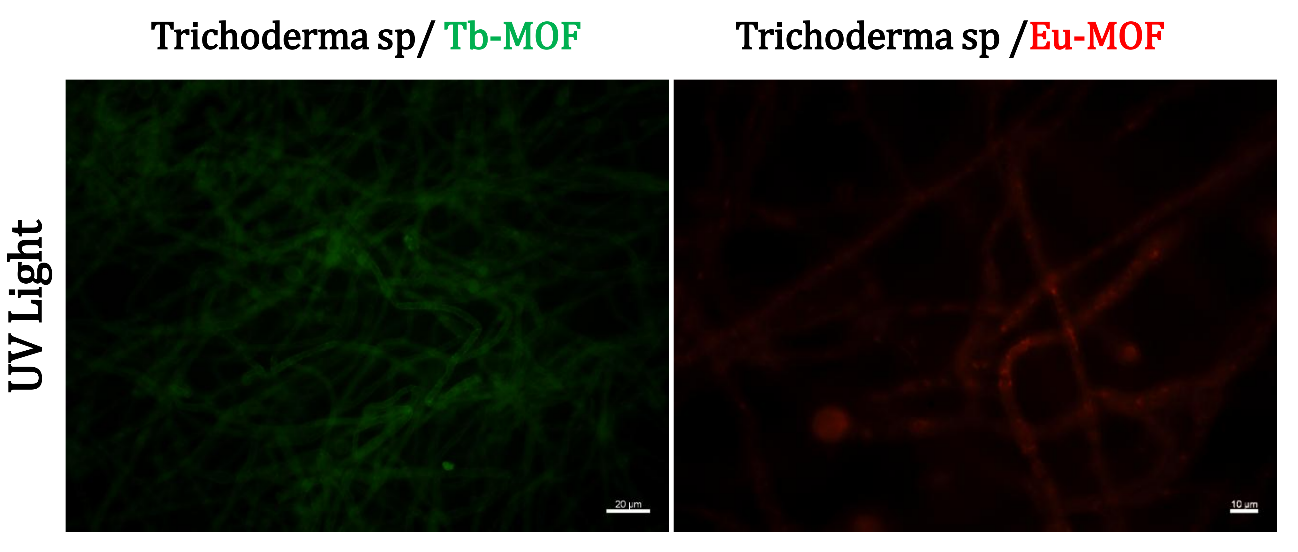


**Figure S11:** Fluorescence images for isolated *Trichoderma sp./*Ln-MOFs hyphae bio-hybrids. (Rosário, J.; da Luz, L.L. *et al.*)


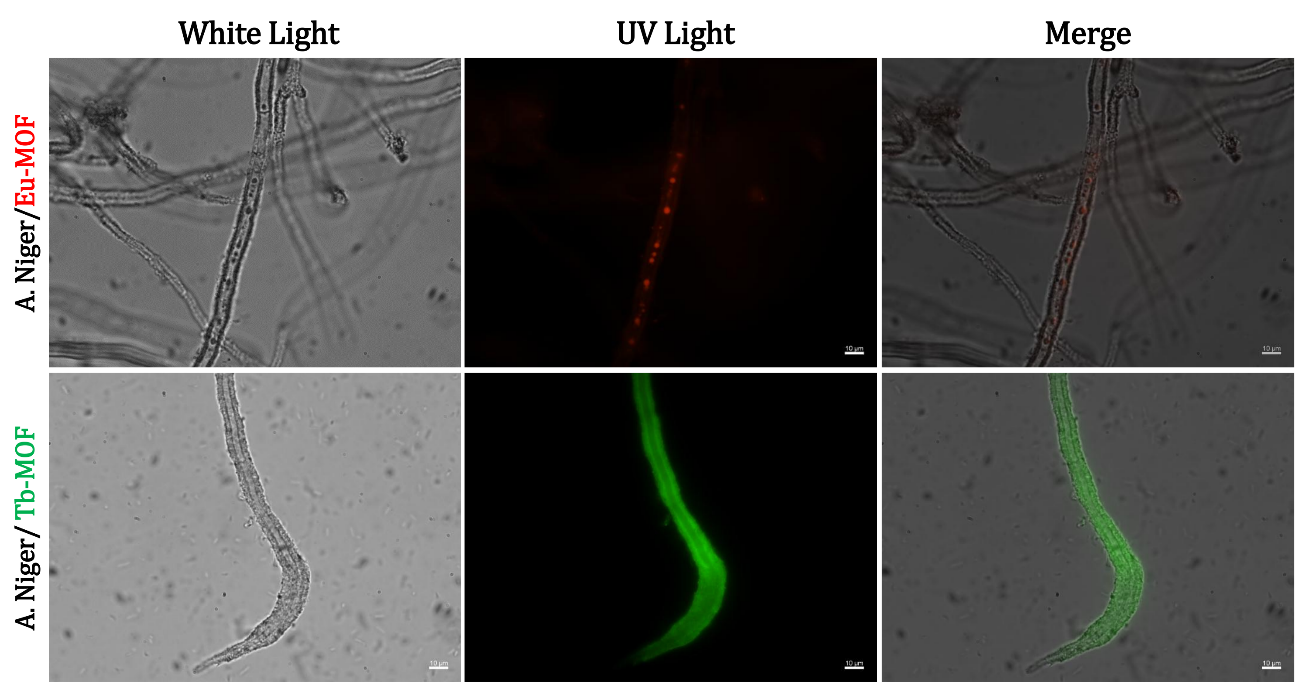


**Figure S12:** Bright field, fluorescence and composite images for isolated *A. niger*/Ln-MOFs hyphae bio-hybrids. (Rosário, J.; da Luz, L.L. *et al.*)


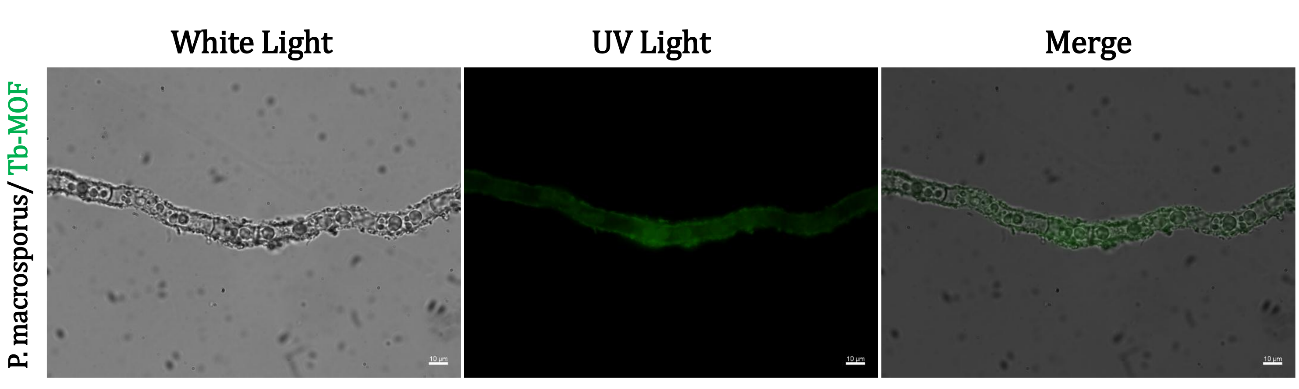


**Figure S13:** Bright field, fluorescence and composite images for isolated *P. macrosporus*/Tb-MOF hyphae bio-hybrid. (Rosário, J.; da Luz, L.L. *et al.*)


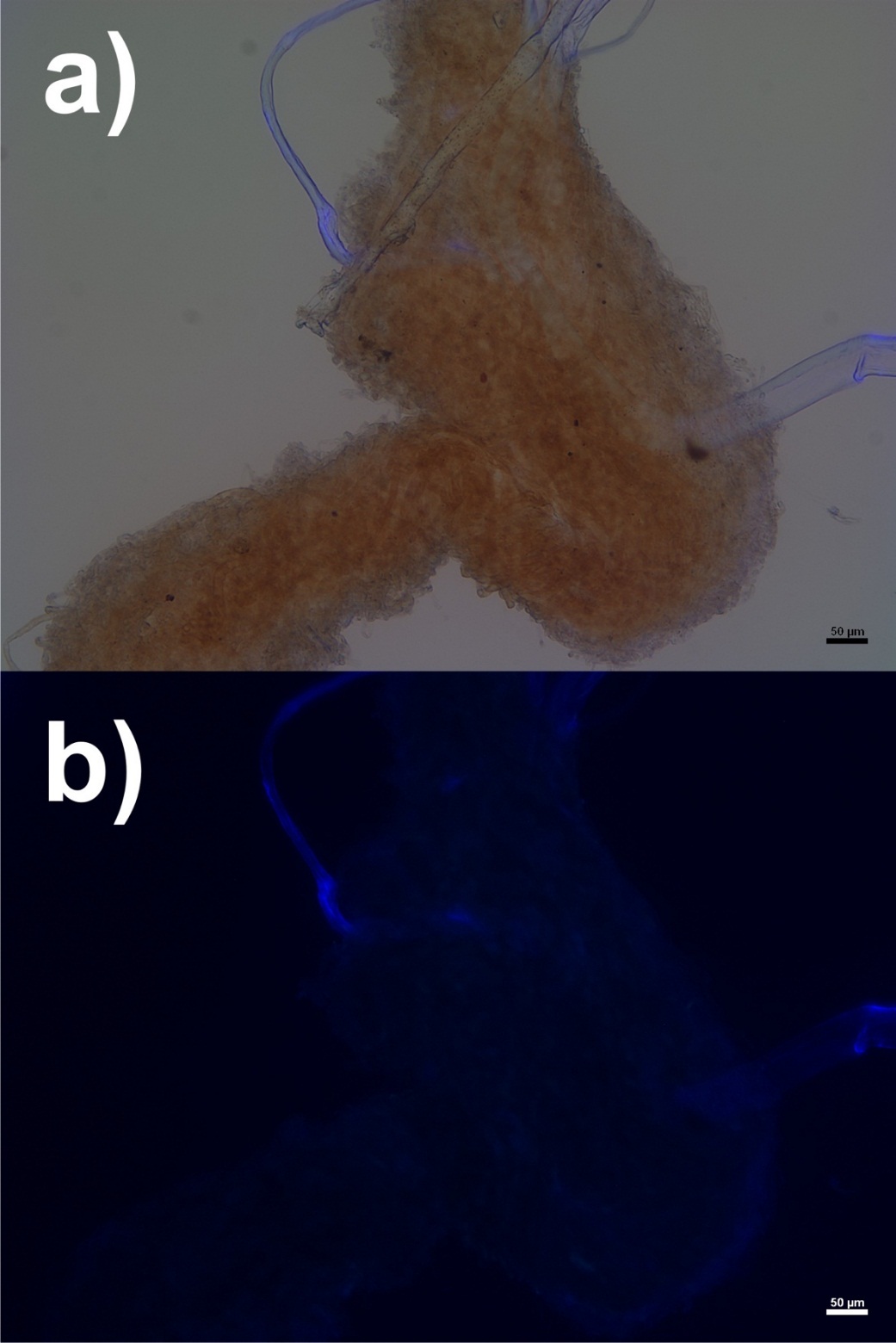


**Figure S14:** (a) Composite and (b) fluorescence images of *P. macrosporus* cultivated in a solution containing Tb(NO_3_)_3_. (Rosário, J.; da Luz, L.L. *et al.*)


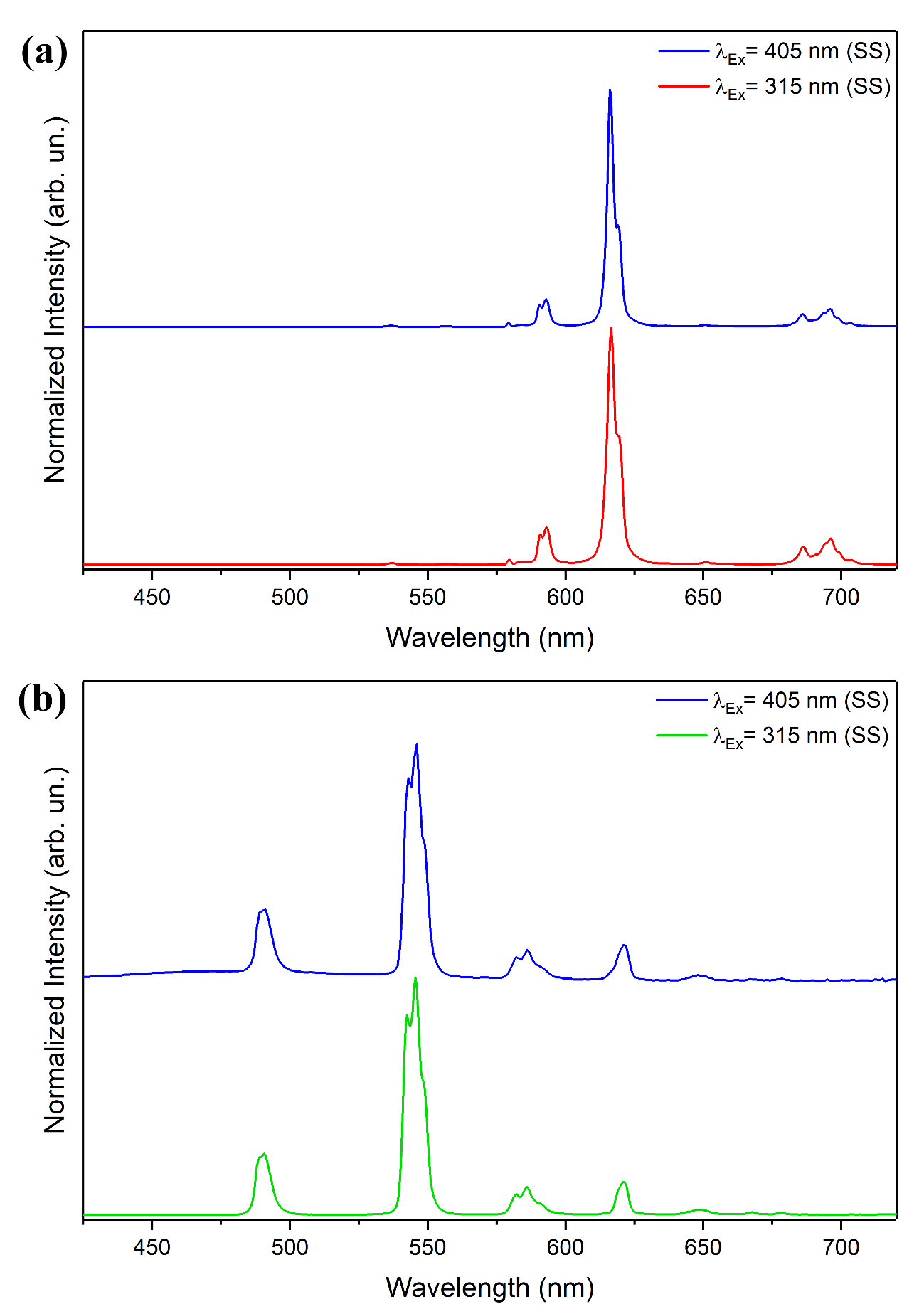


**Figure S15:** Emission spectra of (a) Eu-MOF and (b) Tb-MOF acquired upon excitation at 315 and 405 nm. (Rosário, J.; da Luz, L.L. *et al.*)


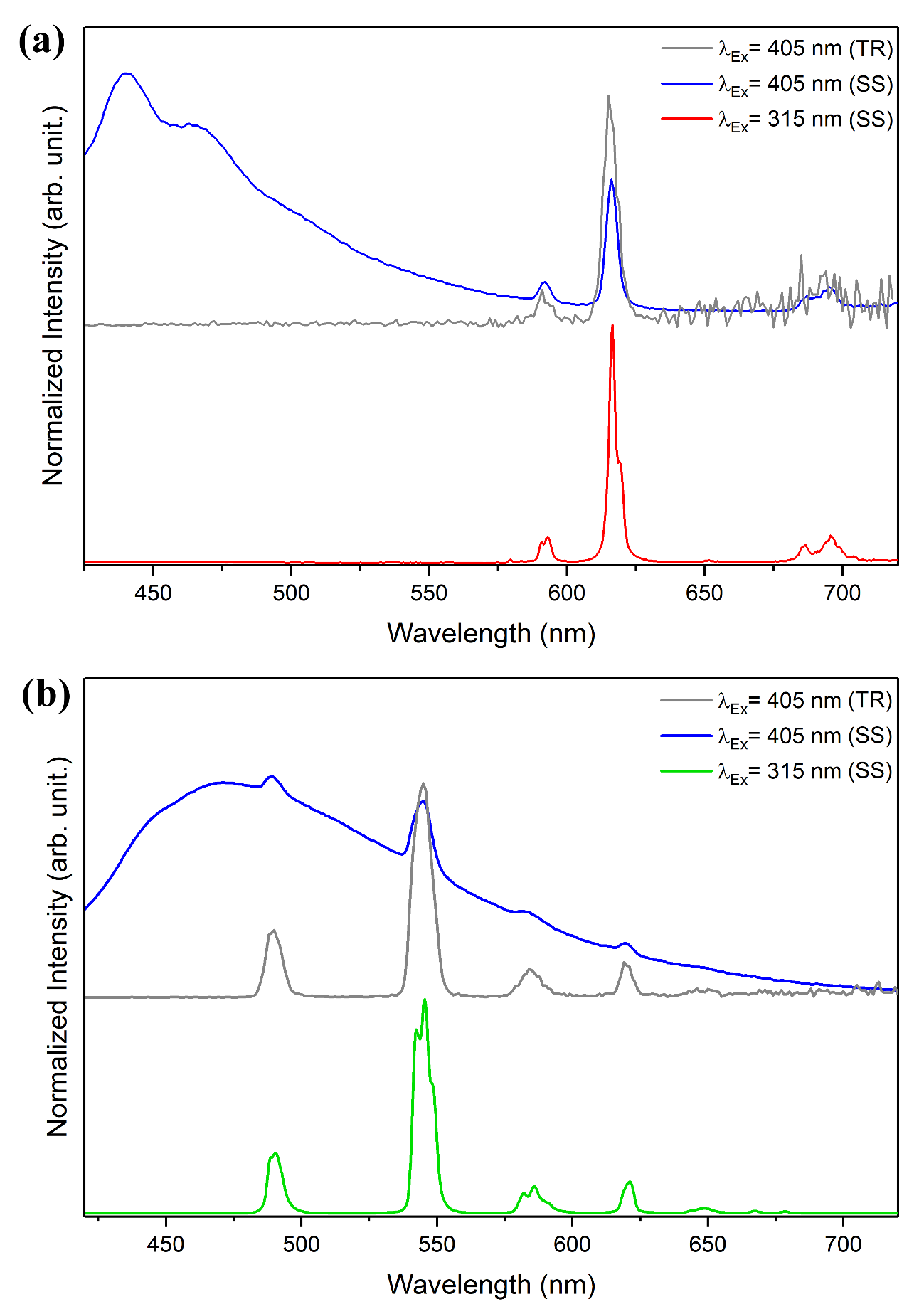


**Figure S16:** Steady-state (SS) and time-resolved (TR) emission spectra of *A. niger*/Eu-MOF (a) and *A. niger*/Tb-MOF (b). For acquisition of the time-resolved spectra, the flash delay was 0.05 ms, with sample window of 0.2 ms, and 100 flash counts were considered. (Rosário, J.; da Luz, L.L. *et al.*)

**

**

**Figure S17:** Experimental XRD pattern of *Trichoderma sp.*/EuTb-MOF and *P. macrosporus*/EuTb-MOF, and calculated XRD pattern of Ln-MOFs. (Rosário, J.; da Luz, L.L. *et al.*)


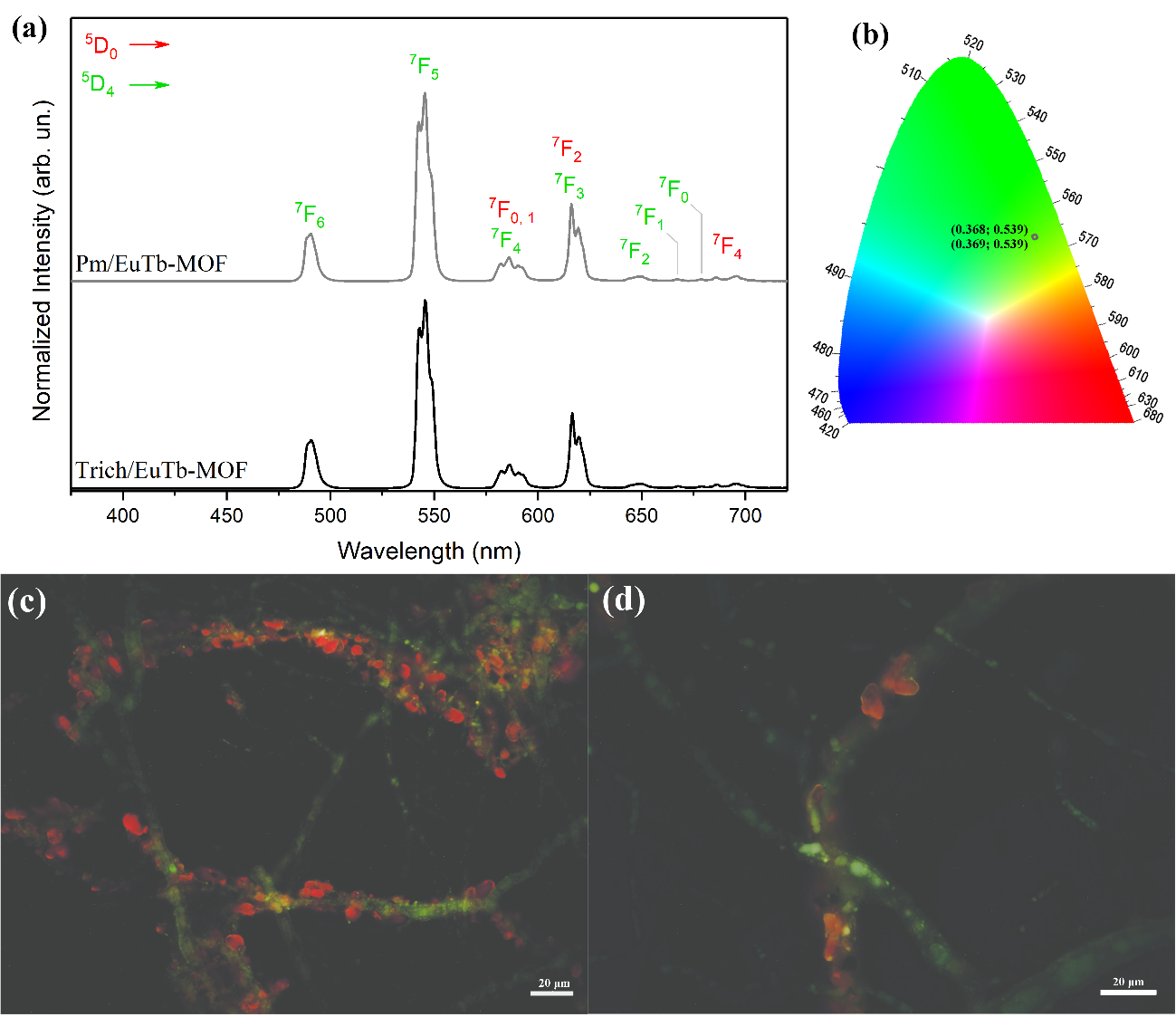


**Figure S18:** (a) Emission spectra of *P. macrosporus*/EuTb-MOF and *Trichoderma* sp./EuTb-MOF acquired upon excitation at 315 nm. (b) Chromaticity diagram is exhibiting the color-coordinated emission of Pm/EuTb-MOF and Trich/EuTb-MOF biohybrids under excitation at 315 nm. Fluorescence images of *P. macrosporus* biohybrids cultivated in a solution containing mixed Tb and Eu-MOF particles at (c) 40X and (d) 100X magnifications. (Rosário, J.; da Luz, L.L. *et al.*)

**
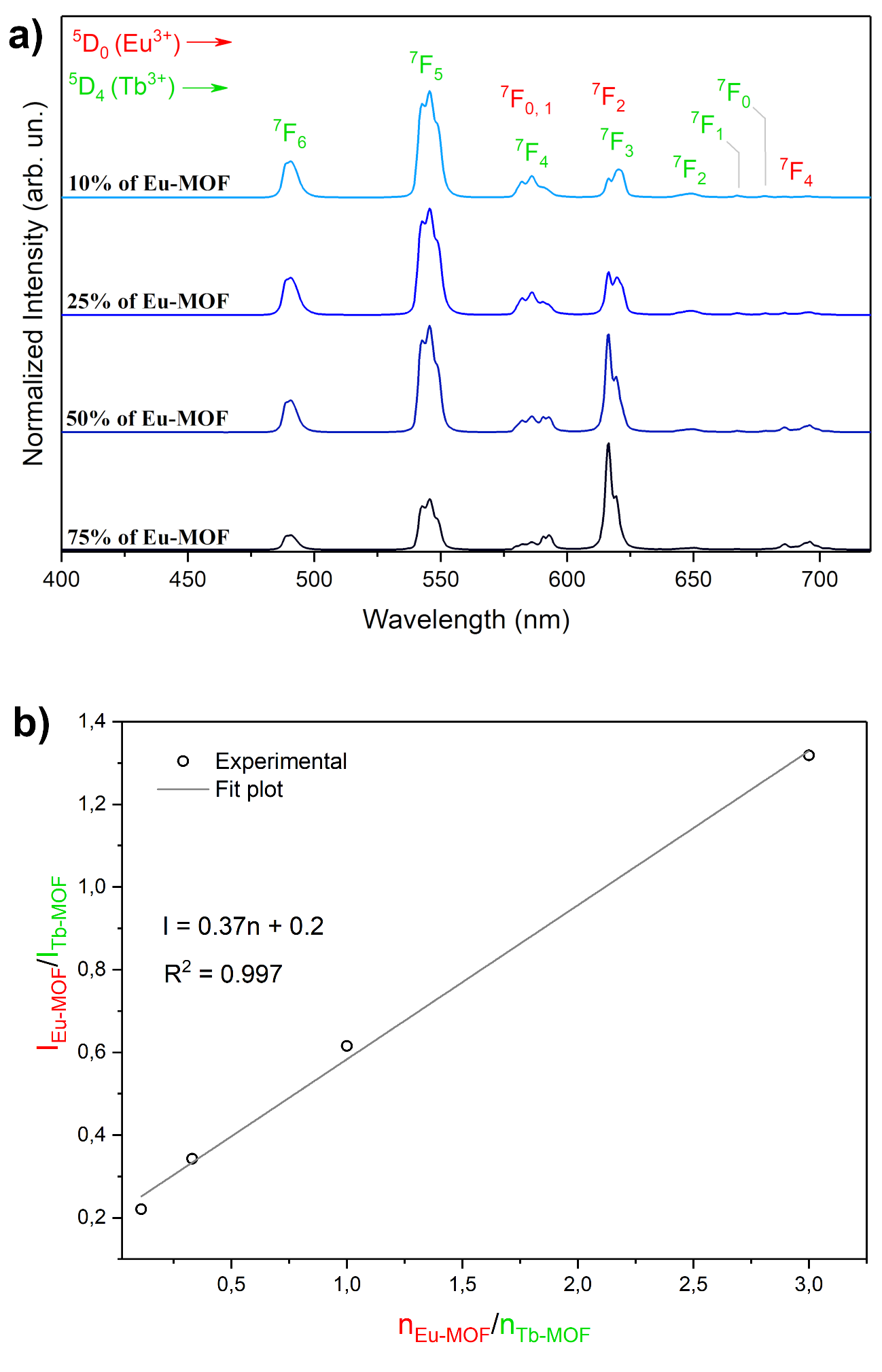
**

**Figure S19:** **(a)** Emission spectra of mixed Eu-MOF:Tb-MOF in a molar ratios of 1:9 (10% of Eu-MOF), 1:3 (25% of Eu-MOF), 1:1 (50% of Eu-MOF) and 3:1 (75% of Eu-MOF) upon excitation at 315 nm. (b) Linear relationship between the intensity ratios of the transitions ^5^D_0_ → ^7^F_2_ (Eu^3+^) / ^5^D_4_ → ^7^F_5_ (Tb^3+^) and the molar ratio n(Eu-MOFs) / n(Tb-MOFs) (Rosário, J.; da Luz, L.L. *et al.*)

**

**

**Figure S20:** Absorption spectra of solutions containing amino acids. (Rosário, J.; da Luz, L.L. *et al.*)

**
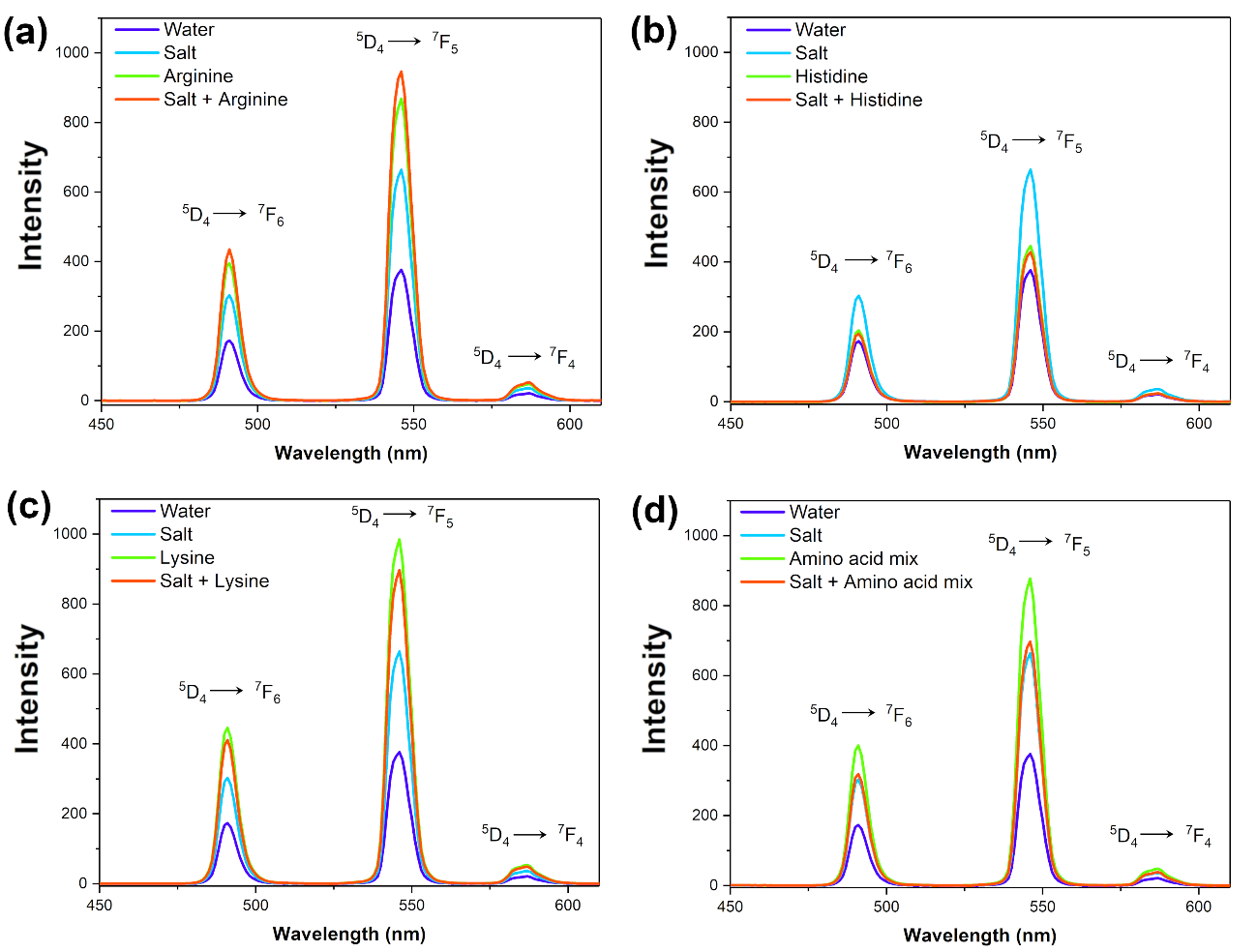
**

**Figure S21:** Emission spectra of Tb-MOF in solutions with presence and absence of amino acids and salts upon excitation at 315 nm: (a) arginine and salts, (b) histidine and salts, (c) lysine and salts and (d) mixture of amino acids and salts. Excitation at 315 nm. (Rosário, J.; da Luz, L.L. *et al.*)

**

**

**Figure S22:** Emission spectra of Eu-MOF in solutions with presence and absence of amino acids and salts upon excitation at 315 nm. (Rosário, J.; da Luz, L.L. *et al.*)

**References**

[1] S. Alves, F. V. deAlmeida, G. F. deSa, C. D. Donega, *Journal of Luminescence* **1997**, *72-4*, 478-480.

[2] K. Binnemans, *Coordination Chemistry Reviews* **2015**, *295*, 1-45; G. F. de Sa, O. L. Malta, C. D. Donega, A. M. Simas, R. L. Longo, P. A. Santa-Cruz, E. F. da Silva, *Coordination Chemistry Reviews* **2000**, *196*, 165-195.
